# Connecting the dots: investigating the link between environmental, genetic, and epigenetic influences in metabolomic alterations in oral squamous cell carcinoma

**DOI:** 10.1186/s13046-024-03141-5

**Published:** 2024-08-21

**Authors:** Ishita Gupta, Fariba Badrzadeh, Yuri Tsentalovich, Daria A. Gaykalova

**Affiliations:** 1 Institute for Genome Sciences, University of Maryland School of Medicine, Baltimore, MD USA; 2 Department of Otorhinolaryngology-Head and Neck Surgery, Marlene & Stewart Greenebaum Comprehensive Cancer Center, University of Maryland Medical Center, Baltimore, MD USA; 3 International tomography center CB RAS, Institutskaya str. 3a, Novosibirsk, 630090 Russia; 4 Department of Oncology, Sidney Kimmel Comprehensive Cancer Center, Johns Hopkins University, Baltimore, MD USA; 5 Institute for Genome Sciences, 670 West Baltimore Street, Baltimore, MD 21201 USA

**Keywords:** Metabolomics, Metabolism, Oral squamous cell carcinoma, Risk factors, Epigenetics

## Abstract

Oral squamous cell carcinoma (OSCC) accounts for around 90% of all oral cancers and is the eighth most common cancer worldwide. Despite progress in managing OSCC, the overall prognosis remains poor, with a survival rate of around 50–60%, largely due to tumor size and recurrence. The challenges of late-stage diagnosis and limitations in current methods emphasize the urgent need for less invasive techniques to enable early detection and treatment, crucial for improving outcomes in this aggressive form of oral cancer. Research is currently aimed at unraveling tumor-specific metabolite profiles to identify candidate biomarkers as well as discover underlying pathways involved in the onset and progression of cancer that could be used as new targets for diagnostic and therapeutic purposes. Metabolomics is an advanced technological approach to identify metabolites in different sample types (biological fluids and tissues). Since OSCC promotes metabolic reprogramming influenced by a combination of genetic predisposition and environmental factors, including tobacco and alcohol consumption, and viral infections, the identification of distinct metabolites through screening may aid in the diagnosis of this condition. Moreover, studies have shown the use of metabolites during the catalysis of epigenetic modification, indicating a link between epigenetics and metabolism. In this review, we will focus on the link between environmental, genetic, and epigenetic influences in metabolomic alterations in OSCC. In addition, we will discuss therapeutic targets of tumor metabolism, which may prevent oral tumor growth, metastasis, and drug resistance.

## Introduction

Oral cancers comprise 75% of the head and neck cancers, of which approximately 90% are oral squamous cell carcinomas (OSCC) [1]. OSCC develops in the oral mucosa and lip [2] and is the eighth most common malignancy worldwide, representing approximately 2% of all malignancies and 1.8% of cancer deaths, with varying occurrences; the highest prevalence is in Asia, followed by Europe and North America [3]. Research studies report a higher prevalence in males than in females, with increasing age as a risk factor [4].

Risk factors for OSCC include tobacco use, alcohol consumption, betel quid chewing, chronic inflammation, human papillomavirus (HPV) infection, diet, immunosuppression, and genetic susceptibility [5, 6]. Tobacco use and alcohol consumption are the most significant risk factors, increasing the risk of developing OSCC by 6-fold and account for up to 75% of all OSCC cases [7]. Moreover, oral inflammation is frequently induced by several molecular pathways associated with inflammation, including nuclear factor kappa-light-chain-enhancer of activated B cells (NF-kB), STAT (signal transducer and activator of transcription), epidermal growth factor receptor (EGFR), p38a MAP kinase, cyclooxygenase-2 (COX-2) and RhoC [8]. Exposure to chemicals like asbestos and benzene, poor oral hygiene, ill-fitting dentures, and ultraviolet (UV) radiation also play a role in the onset and progression of OSCC. OSCC undergoes a multifaceted progression characterized by the accrual of genetic and epigenetic modifications in key regulatory genes. OSCC development often begins with alterations in the normal mucosa, leading to dysplasia, characterized by abnormal cell growth and precancerous changes [9]. Subsequently, cells may progress to carcinoma in-situ, where cancerous alterations are confined to the epithelial layer [9]. As the disease advances, invasive cancer develops, involving the penetration of cancer cells beyond the basement membrane into surrounding tissues [5]. The final and most critical stage is metastasis; OSCC can metastasize to the regional lymph nodes, distant organs (lungs, liver, and bones), as well as the jawbones or facial structures [10].

The diagnosis of OSCC involves a combination of methods, including clinical examination, Toluidine blue staining, and various invasive techniques such as biopsy (incisional, excisional, punch, core needle, or fine needle aspiration). Additional diagnostic tools include exfoliative cytology and routine dental radiographs [10–12]. Although clinical examination determines the clinical staging, imaging with magnetic resonance imaging (MRI), computed tomography (CT) and/or CT- Positron Emission Tomography (PET) is common to determine the disease’s extent, including nodal and distant metastases, thereby enhancing the precision of staging [13]. More than half of OSCC cases are diagnosed at an advanced stage (stages III or IV) as the majority of the patients are asymptomatic in the early stages and delay medical attention until the presence of symptoms such as pain, bleeding, or presence of a lump in the mouth or neck [14]. The management of OSCC is based on the stage of the cancer and typically involves surgical removal of the tumor, followed by radiation therapy and/or chemotherapy [15]. However, postoperative complications of OSCC include problems in chewing, swallowing, dysarthria, and facial disfigurement, which can markedly worsen the quality of life of the patients. Technological progress in the field of molecular biology and genomics has been a driver that has led to the creation of new diagnostic and therapeutic agents like cetuximab (targeting EGFR), as well as nivolumab and pembrolizumab (both blocking PD-1/PD-L1 pathways), with pembrolizumab being particularly effective for treating cancers with a high level of microsatellite instability(MSI-H) OSCC [16, 17]. Nevertheless, the scarce presence of MSI-H in oral cancer hampers the effectiveness of pembrolizumab [18]. Recently, entrectinib, which targets the neurotrophic receptor tyrosine kinase (NTRK) fusion gene, has been approved by the FDA for solid tumors [19]. However, as compared to other cancers, the availability of molecularly targeted drugs for OSCC remains limited [20]. Although there have been improvements in the management of OSCC, the prognosis for OSCC is still poor, with an overall survival rate of around 50–60% due to metastasis [21]. Due to the various difficulties associated with current diagnostic approaches and the typically late diagnosis of OSCC, there is a critical demand for the development of less invasive techniques that can identify and manage the disease at earlier stages, thus potentially improving patient outcomes. Furthermore, suggesting alternative drug-based therapies that can serve as effective substitutes for conventional treatments as mentioned above would be highly beneficial. Enhanced comprehension of metabolic changes in OSCC could potentially advance early diagnosis and prognostic predictions through the application of metabolomic analysis.

Cancer cells are known to depend on aerobic glycolysis for generating adenosine 5′-triphosphate (ATP) and metabolic intermediates [22]. A common characteristic of this altered metabolism is the heightened uptake of glucose and its conversion to lactate, a phenomenon known as the “Warburg effect” [22]. The cancer cells undergo metabolic rewiring to support growth, survival, proliferation, and long-term maintenance [23]. This metabolic adaptation is geared towards facilitating the assimilation of nutrients into biomass, including nucleotides, amino acids, and lipids, essential to produce new cells [24]. In head and neck squamous cell carcinoma (HNSCC), aerobic glycolysis is the main source of energy supply for the tumor [25].

Metabolomics is one of the emerging ‘omic’ sciences that seek to explore the molecular complexity of life. These approaches aim to enhance our understanding of biological systems at the molecular level, particularly when integrated with genomics and proteomics data within the framework of systems biology [26]. Metabolomics utilizes advanced analytical instrumentation along with pattern recognition techniques (mass spectrometry and nuclear magnetic resonance spectroscopy) to analyze the metabolome, encompassing all small molecules (50–1500 Da) with varied physiochemical traits and a dynamic range of abundance, commonly referred to as metabolites [27]. Study of the metabolome can be conducted in cells, biofluids, or tissues, revealing molecular phenotype through the identification of metabolites and their concentrations influenced by environmental factors, genetic variations, or alterations in the microbiome directly.

Depending on their role and origin in metabolism, metabolites can be classified into two major categories: primary and secondary metabolites. Primary metabolites (amino acids, nucleotides, and carbohydrates) are essential for the basic maintenance of life processes and are involved in fundamental cellular functions like energy production, growth, and reproduction [28]. Unlike primary metabolites, secondary metabolites are not directly involved in these core processes but often contribute to an organism’s adaptation to its environment. Secondary metabolites (alkaloids, flavonoids, antibiotics, pheromones, pigments, and terpenoids) often play roles in defense mechanisms or serve as signaling molecules [29]. Like other omic technologies, metabolomics is presently employed for identifying biomarkers and altered metabolic pathways in cancer [30–32]. Since cancer significantly influences cellular metabolism, metabolomics can play a pivotal role in the early detection, diagnosis, and evaluation of medical interventions for cancer [32].

This review aims to summarize and explore the existing studies on metabolomics in OSCC. We will delve into the various technological tools employed in metabolomics involved in the identification and characterization of metabolites that participate in the pathogenesis of OSCC and concentrate on findings from studies utilizing diverse patient samples (e.g., saliva, serum, blood, urine, and tissues). Further, we will report how the dysregulation of these metabolites may be associated with environmental, genetic, and epigenetic factors influencing the disease. By integrating metabolomics data with other omics information, a more comprehensive understanding of intricate cancer processes can be achieved. This interdisciplinary approach may yield novel insights to better target aggressive and malignant cancer types, including OSCC. On the other hand, although the study concerning tumor relapse and the characterization of metabolomics’ role in the comprehension of drug-tolerant persisted cancer cells are indeed critical, they are not the main subject of our review. The overall objective of this narrative review is to emphasize the promise of metabolomic profiling as a suitable modality for advancing early detection, timely intervention, and, ultimately, better patient outcomes for initial OSCC diagnosis. However, any such in-depth analysis focusing on metabolic alterations linked to tumor recurrence is a question of its own and beyond the scope of this review.

## Metabolism in oral cancer

Metabolism involves a set of biochemical reactions that convert nutrients into metabolites, facilitating the generation of energy crucial for cell survival [33]. Under normal conditions, absorbed nutrients undergo glycolysis, followed by the mitochondrial tricarboxylic acid (TCA) cycle coupled with oxidative phosphorylation (OxPhos), producing adenosine 5′-triphosphate (ATP) to fulfill cellular energy needs [23]. On the contrary, during tumorigenesis, cancer cells alter these metabolic pathways to generate the requisite energy for increased cell proliferation and migration [33]. In the following sections, we will review the metabolic reprogramming underpinning the onset and progression of OSCC cells.

### Glucose metabolism

As exemplified by the Warburg effect, cancer cells undergo significant metabolic alterations, a fundamental characteristic showcasing heightened aerobic glycolysis [22]. Cancer cells with an increased glycolytic phenotype exhibit elevated glucose consumption and oxidation, converting glucose into pyruvate with concurrent lactate production [22]. This metabolic process involves the upregulation of various glycolytic enzymes, including pyruvate kinase M2 (PKM2), hexokinase 2 (HK2), phosphofructokinase (PFK), and glucose-6-phosphate dehydrogenase (G6PD); overexpression of these enzymes was observed in OSCC and is associated with the prognosis of OSCC patients [34–38]. Additionally, in OSCC, glycolysis addiction is apparent through increased glucose uptake, primarily indicated by the upregulation of glucose transporter protein (GLUT), particularly GLUT1 and GLUT3 [39, 40]. Studies have reported elevated expression of these transporters to significantly correlate with a poor prognosis in OSCC patients [40–43]. Notably, the Warburg effect has significant implications for therapies targeting HNSCC. Beyond traditional chemotherapy, cetuximab, an anti-EGFR monoclonal antibody, counteracts the Warburg effect by inhibiting HIF-1α regulated lactate dehydrogenase A (LDH-A), thus, disrupting the metabolic reprogramming that promotes cancer cell survival [44]. However, cancer cells can adapt to this metabolic disruption by rewiring through mechanisms including the activation of acetyl-coenzyme A (acetyl-CoA) carboxylase [45]. Nevertheless, this adaptation underscores the need for comprehensive treatment strategies. On the other hand, the Warburg effect’s influence extends to immunotherapy, where it can modulate the immune response. Peng et al. (2016) [46] demonstrated that aerobic glycolysis enhances T helper 1 (Th1) cell differentiation *via* epigenetic mechanisms, with LDH-A maintaining high concentrations of acetyl-CoA to trigger histone acetylation and transcription of IFN-γ. Additionally, in vivo data reported the ablation of LDH-A in T-cells to protect mice from immunopathological responses triggered by high IFN-γ expression of lack of regulatory T-cells [46]. The study revealed an epigenetic mechanism by which aerobic glycolysis promotes effector T-cell differentiation, thus indicating LDH-A may be targeted therapeutically and manipulation of T cells’ metabolic pathways can enhance immunotherapeutic outcomes [46]. By targeting the metabolic vulnerabilities of both cancer and immune cells, novel therapeutic approaches can improve the efficacy of treatments like cetuximab and immunotherapy.

In contrast, non-glycolytic (OxPhos) cancer cells generate lactate from pyruvate triggered by oxidative stress induced by molecules released by cancer-associated fibroblasts, as even noted in OSCC [47]. Subsequently, non-glycolytic cancer cells metabolize the produced lactate through the TCA cycle, a phenomenon referred to as the “reverse Warburg effect” [48]. The enzyme lactate dehydrogenase (LDH), responsible for converting pyruvate to lactate, was found in the serum and saliva of OSCC patients; analysis of LDH can aid in the detection and diagnosis of OSCC [49–51]. Additionally, analyzing LDH expression in tumor tissues proved valuable in predicting patient prognosis and responses to chemotherapy [52–54].

### Lipid metabolism

Lipid metabolism involves the synthesis, breakdown, storage, and transportation of various types of lipids in the body, giving rise to a range of bioactive lipid molecules [55]. The upsurge in lipid metabolism represents a distinct characteristic of cancer metabolism, where disruptions or irregularities in these signaling pathways may lead to aberrant cell proliferation and growth [56]. Studies have reported dysregulation of lipid metabolism in OSCC; altered lipid metabolism genes were associated with clinical features and patient prognosis [57, 58]. Cancer cells actively engage in the uptake and synthesis of fatty acids (FAs) via the low-density lipoprotein receptor, CD36, FA transporter proteins, and FA binding proteins (FABPs). These processes contribute to the metabolic dynamics within cancer cells. Studies on CD36 in OSCC have revealed its correlation with OSCC progression, proliferation, migration, and lymph node metastasis [59, 60]. Halczy-Kowalik et al. (2019) [61] analyzed the FA content of the serum, tumor tissue, and adjacent tumor microenvironment by gas chromatography in OSCC patients (grade 1–3) and revealed 19 FAs in tumor tissue, tumor-adjacent tissue, and blood serum. The study further reported correlations between FA and grade 1 + 2 tumors, with a significant association with grade 3 tumors, thus indicating a differential role of FAs in the metabolism of lower (grade 1 + 2) and higher grade (grade 3) tumors [61]. FABPs − 4 and − 5 were also reported to stimulate OSCC cell growth, invasion, and migration [62, 63]. Fatty acid synthase (FASN) is vital for lipid synthesis and is associated with oncogenic activity in various cancers. In OSCC, overexpression of FASN promotes cell proliferation and migration as well as chemotherapy resistance [64], thus, correlating advanced disease and an unfavorable prognosis [65, 66]. The FA derivative compound, Prostaglandin E2 (PGE2) is produced by cyclooxygenase (COX)-2; both PGE2 and COX-2 stimulate OSCC invasion and proliferation [67, 68]. Cholesterol metabolism was also reported in OSCC with elevated cholesterol levels promoting oral carcinogenesis [69–71].

During lipid metabolism, oxidative stress produces free radicals, particularly reactive oxygen species (ROS), which trigger oxidative degradation of lipids, especially PUFA, within cell membranes, resulting in the formation of lipid peroxides and subsequent cellular damage [72, 73]. This process, known as lipid peroxidation, produces various reactive aldehydes such as malondialdehyde (MDA), 4-hydroxynonenal (4-HNE), propanal, and hexanal [74–76], of which MDA is considered as the most mutagenic product [77] while 4-HNE is recognized as highly cytotoxic [78]. These secondary products can induce cellular damage, leading to ferroptosis, an iron-dependent regulated necrosis [79, 80]. Given the complex interplay of oxidative stress and antioxidant defense mechanisms demonstrated in several investigations in OSCC patients, lipid peroxidation is thought to represent a central part of the pathophysiological mechanisms involved. Enhanced levels of lipid peroxides, especially MDA, have been consistently detected in the saliva and serum of patients with OSCC compared to healthy subjects [81–83], indicating an increase of oxidative damage in these patients. This oxidative stress along with simultaneous decrease in the antioxidant enzyme activities such as superoxide dismutase (SOD), glutathione peroxidase (GPx), and glutathione (GSH), as well as lower total thiols and antioxidant vitamins such as vitamins E and C, further increases cellular damage [84–88]. Likewise, another study also reported increased MDA levels and lower vitamin A and C levels in the plasma of oral cavity and oropharyngeal cancer patients as compared to healthy controls [89]. Manohar and colleagues [85] used colorimetric assays and reported increased lipid peroxidation levels and a marked reduction in both non-enzymatic and enzymatic oxidant status as compared to healthy subjects. The study further showed that while levels of thiobarbituric acid reactive substances (TBARS) progressively increased, antioxidant levels consistently decreased from stage II to stage IV of oral cancer patients [85], thus indicating the imbalance between lipid peroxidation and antioxidant defenses are implicated in the progression from precancerous lesions (leukoplakia and oral submucous fibrosis) to malignant stages. Studies have shown increased lipid peroxide levels and reduced antioxidant status to correlate with poor overall survival in OSCC patients [90], thus indicating the potential of MDA and TBA as biomarkers in prognostication. In addition, a recent study reported resistance of oral cancer cells to ferroptosis, thus offering a novel therapeutic approach [91].

Notably, research has shown targeting lipid peroxidation can aid in cancer treatment. Persister cells, a subset of cancer cells that develop drug resistance by entering a quiescent state, can alter redox homeostasis and stimulate antioxidant defenses, further mitigating the effects of lipid peroxidation, thus allowing the cells to evade apoptosis [92]. Persister cancer cells were reported to be vulnerable to inhibition of GPx4, leading to enhanced lipid peroxidation and ferroptosis [93], thus suggesting the potential of targeting lipid peroxidation pathways to overcome drug resistance in cancer cells. In head and neck cancer as well, activation of the Nrf2-antioxidant response element (ARE) pathway led to ferroptosis resistance in cisplatin-resistant head and neck cancer cells [94]. However, combined therapy targeting the Nrf2-ARE pathway with artesunate effectively eliminated this resistance [94]. The data from the above studies suggest the strategic use of stimulating lipid peroxidation and inhibiting antioxidant pathways to enhance cancer cells’ sensitivity to treatment and over-drug resistance, thereby improving clinical outcomes.

Additional investigations are needed to validate the increased activity of lipid metabolism, including lipid peroxidation in OSCC development and progression, highlighting candidate avenues for diagnostic, prognostic, and therapeutic interventions.

### Amino acid (AA) metabolism

Tumor cells exhibit heightened reliance on external non-essential amino acids and demonstrate elevated engagement in amino acid synthesis, degradation, and transportation [95]. This dependence on amino acids is attributed to their roles in energy provision, redox balance regulation, and support for protein and lipid synthesis [95].

Glutamine (Gln), a non-essential AA, serves as the ancillary principal nutrient for the survival of many tumor cells [96]. Gln entry into the cancer cells is facilitated by 14 AA transporters, such as the Na+-dependent system ASC (alanine/serine/cysteine-preferring) and the Na+-coupled neutral amino acid transporters (SNATs) from the SLC38 superfamily [97]. Among these, SLC1A5/ASCT2 is overexpressed in OSCC and stimulates cell growth and proliferation [98, 99]. Glutaminolysis (glutamine catabolism) involves the conversion of Gln to glutamate *via* glutaminase (GLS) [100]. Studies have reported the upregulation of GLS in OSCC, which is associated with poor prognosis [101, 102]. In addition, overexpression of asparagine synthetase (ASNS) is responsible for transferring an amide group from Gln to aspartate to facilitate asparagine formation [103]. Fu et al. (2021) [104] reported a significant association between the overexpression of ASNS and lymph node metastasis and perineural invasion in OSCC, thus indicating ASNS as a prognostic marker.

In serine/glycine metabolism, serine hydroxymethyltransferase (SHMT1 and SHMT2 in the cytoplasm and mitochondria, respectively) facilitates the transfer of the beta carbon of serine to tetrahydrofolate (THF), resulting in the formation of glycine and one-carbon units (5,10-methylene-THF), a crucial process for nucleotide synthesis [105]. While one-carbon metabolism takes place in both the cytoplasm and mitochondria, only SHMT2 is overexpressed and correlates with tumor aggressiveness and prognosis [106, 107]. A very recent study indicated that a transition from the uptake of external serine to the internal synthesis of serine promotes the differentiation of OSCC cells [108]. Similarly, Liao et al. (2021) [109] revealed upregulation of SHMT2 in OSCC significantly enhanced cell proliferation, cell cycle progression, invasion, and migration and correlated with an unfavorable prognosis.

Apart from the AAs mentioned above, OSCC may depend on other AAs, including methionine [110, 111], arginine [112, 113], as well as AA transporters (L-type amino acid transporter 1) [114].

In the following sections, we will review how the risk factors affect OSCC cellular metabolism. Finally, we will review the application of metabolomics in clinical and translational research, offering insights into potential future applications.

## Factors affecting metabolism in OSCC

### Environmental factors

**Fig. 1:**
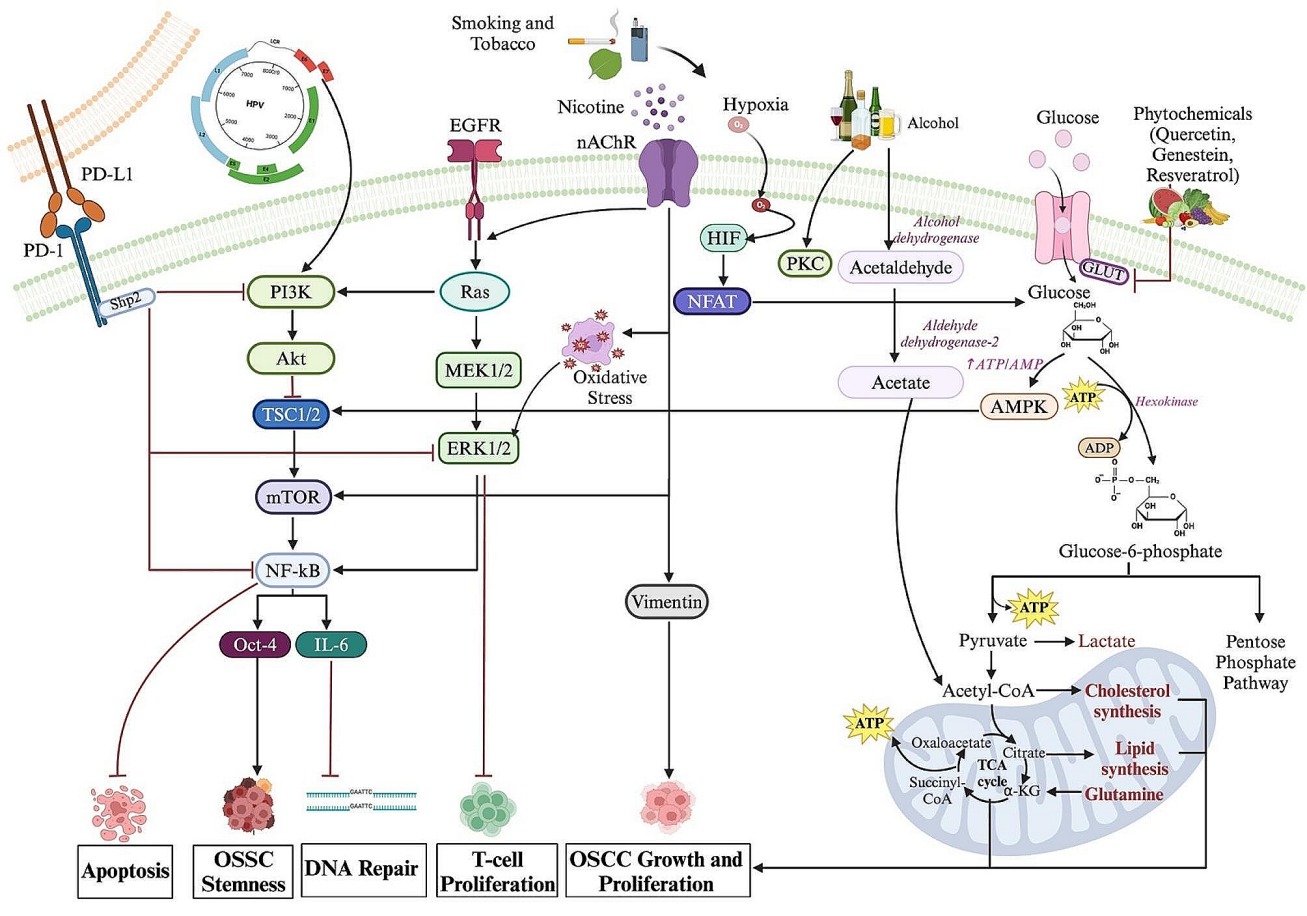
The intricate interplay of environmental, genetic, and epigenetic factors in OSCC metabolism. This schematic illustration highlights the pivotal role of the different components in shaping the metabolic landscape of oral cancer. External elements such as tobacco smoke, alcohol consumption, and exposure to carcinogens modulate key metabolic pathways, including glycolysis and oxidative phosphorylation. Various components of the diet, including nutrients and bioactive compounds, influence crucial metabolic pathways. Genetic alterations also influence key metabolic pathways, impacting cellular processes such as glycolysis, tricarboxylic acid (TCA) cycle, and nucleotide metabolism. The intricate interplay between genetic and environmental influences shapes the metabolic signature of oral cancer, providing insights for comprehensive therapeutic approaches and preventive measures. Understanding the genetic basis of oral cancer metabolism is crucial for developing targeted therapeutic strategies and personalized interventions. α-KG: Alpha-Ketoglutarate; ADP: Adenosine diphosphate; ATP: Adenosine triphosphate; EGFR: Epidermal growth factor; IL-6: Interleukin-6; nAChR: Nicotinic acetylcholine receptor; OSCC: Oral squamous cell carcinoma; TCA: Tricarboxylic acid. Figure created with BioRender.com

As mentioned above, environmental risk factors for OSCC include tobacco use, betel nut chewing, alcohol consumption, poor diet, obesity, and HPV infection [5]. These environmental factors induce DNA damage, alter gene expression, and affect cellular metabolism, with tobacco and alcohol causing oxidative stress and contributing to OSCC development [115, 116] (Fig. 1). Additionally, exposure to certain viruses like human papillomavirus (HPV), particularly HPV type 16 and 18, has been linked to an increased risk of developing HNSCC [117]. The majority of HPV-induced HNSCC comprise oropharyngeal squamous cell carcinomas; however, the role of HPV in OSCC genesis still lies nascent and warrants further investigation [118]. Prusinkiewicz et al. (2020) [119] reported that while HPV − HNSCCs predominantly rely on glycolysis, HPV + HNSCCs exhibit a higher dependence on the TCA cycle, cellular respiration, and β-oxidation. Further research is essential to understand the role of HPV in underpinning OSCC and develop targeted therapies that can effectively address the metabolic dependencies of HPV-related cancers.

#### Tobacco smoking

Tobacco is consumed in various forms, including cigarettes, chewing tobacco, cigars, and hookahs. Tobacco smoking, as well as exposure to secondhand smoke, are vital in contributing to the onset and progression of cancer. It is well established that cigarette smokers are at a significantly higher risk for developing oral cancer in comparison to non-smokers [120]. Tobacco and tobacco smoke contain various carcinogenic compounds like nitrosamines, aromatic amines, and benzopyrenes, which significantly interfere with cellular metabolism, leading to cellular damage in the oral cavity and oropharynx and promoting OSCC development [121, 122].

Alterations in metabolic pathways between smokers and non-smokers, 4-(methylnitrosamino)-1-(3-pyridyl)-1-butanone (NNK)-treated and untreated oral cells, as well as cancerous and noncancerous oral tissue, offer valuable insights into the mechanistic pathways underlying the development of oral cancer. Deregulated pathways involved in the development of tobacco-related oral cancer include amino acid metabolism [95], carbohydrate metabolism [23], fatty acid metabolism [123], nucleotide metabolism [124], oxidative phosphorylation [23], polyunsaturated fatty acid metabolism [125], steroid metabolism [126] and vitamin (A and B) metabolism [127, 128]. A recent metabolomic analysis in oral cancer reported alteration in the above-mentioned pathways, indicating that smoking modifies energy-producing and consuming pathways, potentially promoting oral oncogenesis [129]. Tobacco smoke decreases glucose uptake in OSCC cells and inhibits the activity of the enzyme pyruvate dehydrogenase, thus allowing cancer cells to generate energy in the absence of oxygen [24, 130]. This metabolic shift promotes the growth and survival of OSCC cells by providing them with the energy and substrates necessary for rapid proliferation [130]. Exposure of oral cancer cells to cigarette smoke condensate increases expression of cytochromes P450 and aldo-keto reductases, which are involved in polycyclic aromatic hydrocarbons metabolism, indicating a role of cigarette smoking in altering metabolic processes within these cells [131]. Similarly, another study reported that cigarette smoke extract induces cytotoxicity on oral epithelial cells by increasing oxidative stress [132]. In addition, the study also reported oral cancer progression due to epithelial-mesenchymal transition (EMT) *via* the alteration of the vital players regulating the mitochondrial heme-metabolic pathway [132]. Zhu et al. (2021) [133] reported that smoke triggers glutamine translocators in macrophages, thus increasing the intracellular transport of glutamine, leading to the initiation of glutamine metabolism. Active glutamine metabolism was found to alter the local immune metabolism microenvironment within the oral mucosa and promote abnormal cell proliferation while inhibiting cell apoptosis [133]. Likewise, nicotine, an agonist of the non-selective nicotinic acetylcholine receptor (nAChR), is the main constituent in cigarette smoke. Although not a direct carcinogen, nicotine can induce cancer cell growth, cell proliferation as well as angiogenesis [134]. In oral cancer cells, nicotine exposure enhanced EGFR phosphorylation via α7 nAChR, leading to the activation of the MEK/ERK and PI3K-AKT pathway, thus indicating nicotine exposure to promote oral cancer cell growth and migration [135]. Although studies have reported an association of smoking with oral cancer metabolism, further studies are necessary to validate the role of these smoking-induced metabolic perturbations underlying oral cancer development.

#### Alcohol consumption

Another environmental factor is alcohol consumption, which not only causes direct damage to the cells but also enhances the carcinogenic effect of tobacco [136–138]. Alcohol can induce changes in gene expression and enzyme activity, leading to metabolic alterations that promote OSCC development [139]. Ethanol present in the wine undergoes oxidation in the oral cavity, producing acetaldehyde, a genotoxic metabolite known for its ability to overexpress oncogenes and silence tumor suppressor genes [140]. During the process of carcinogenesis, ethanol and acetaldehyde include alterations in methyl transfer, leading to DNA hypomethylation, altering the expression of oncogenes and tumor suppressor genes, and triggering tumor cell dissemination [140]. Yu and colleagues [141] performed an *in silico* differential expression analysis utilizing RNA sequencing (RNA-seq) in 34 HNSCC patients and reported dysregulation of two significant long non-coding RNAs (lncPSD4-1 and in-NETO1-1) due to alcohol consumption. Moreover, the authors validated the finding in alcohol and acetaldehyde-exposed oral keratinocytes, indicating an association of lncPSD4-1 and in-NETO1-1 with the onset and progression of HNSCC [141]. In vivo studies have demonstrated an alcohol-induced increase in glycolytic enzymes, content of glucose and lactate, and activity of phosphofructokinase [142, 143]. A recent study by Nguyen et al. (2022) [144] reported that chronic alcohol exposure escalates aerobic glycolysis and the stemness phenotype *via* the Nuclear Factor of Activated T Cells (NFAT) signaling pathway, leading to an increase in OSCC cell growth and malignancy. Alcohol-induced NFATc2 expression upregulated the expression of genes involved in glycolysis and cancer stemness (HIF1α, TP1, ENO1, PKM2, ALDH1A, Bmi1, and Oct4) in OSCC [144]. NFAT’s significant role was reported in metabolic reprogramming in different human cancers, influencing glycolysis [145–147].

Several animal models injected with chemical carcinogens are used to study OSCC, and, as compared to the chemical carcinogens 20-methylcholanthrene (20MC), coal tar, and 9,10-dimethyl-1,2-benzanthracene (DMBA), the chemical carcinogen 4-nitroquinoline-1-oxide (4NQO) is commonly used to study OSCC formation [148]. 4NQO, an aromatic amine heterocyclic compound, induces random mutations and forms DNA adducts [149, 150], a crucial mechanism shared between 4NQO and tobacco carcinogens. Additionally, 4NQO also induces intracellular oxidative stress, resembling the effects of tobacco carcinogens [151]. The pathology induced by 4NQO intently mimics the human OSCC model, specifically reflecting OSCC progression at various pathological stages [148]. An in vivo study by Knobloch et al. (2019) [152] using a rat oral cancer model induced by 4NQO identified metabolites associated with glycolysis and AMPK pathways. Similarly, another study reported a substantial metabolic transformation in the 4NQO-induced oral carcinogenesis model, marked by an augmentation in glycolysis and a deficiency in the TCA cycle [153]. Moreover, in murine tongue cancer models, administration of (4NQO) along with alcohol enhanced expression of the active form of β-catenin as compared to 4NQO alone, indicating alcohol triggered the OSCC stemness phenotype [154, 155]. Studies in several cancers highlighted the crucial role of metabolic reprogramming in the genesis and maintenance of cancer stem cell (CSC) populations [156–160]. In comparison to non-CSCs, CSCs express elevated levels of glycolytic enzymes (Glut-1, HK, G6PD, PDK1, PKM2, and LDH) and exhibit heightened aerobic glycolytic activity, triggering CSC population and phenotype [156–163]. Chronic alcohol exposure promotes cancer stemness in OSCC by enhancing aerobic glycolysis [144]. However, the molecular mechanisms underlying these alcohol-induced events still lie nascent. Hence, investigating the impact of chronic alcohol exposure on non-tumorigenic oral keratinocytes would contribute to a broader understanding of alcohol’s effect on OSCC progression.

#### Diet and nutrition

As mentioned above, cancer cells depend on aerobic glycolysis for energy production and the generation of metabolic intermediates [22]. As a result, cancer stimulates the expression of several proteins and enzymes associated with glycolytic metabolism [164].

A quantitative micro dialysis (µD) study in HNSCC patients reported a reduction in lactate concentrations in tumor tissue as compared to the tumor-free mucosa, indicating the use of lactate by the HNSCC cells to generate energy for oxidative glucose metabolism [25]. Similarly, a questionnaire-based study in OSCC reported an association between OSCC and intake of refined carbohydrates [165]. In addition, Ma and colleagues [166] performed a study in preclinical athymic-*nu/nu* female bearing HNSCC cell line xenografts (FaDu) on the flanks, who were fed either standard mouse chow or a glucose-free diet and exposed to ionizing radiation. The study showed that mice with xenografts that received radiation and a glucose-free diet slightly inhibited tumor growth rate and improved survival as compared to those receiving radiation alone [166]. Similarly, a recent in vivo study in OSCC also demonstrated a slight improvement in hamster models when exposed to a glucose-free diet [167]. Ogawa et al. (2014) [168] performed metabolome analysis using capillary electrophoresis and a time-of-flight mass spectrometer to study the metabolic system of OSCC patients and reported aerobic glycolysis in OSCC patients to result from the synergistic augmentation of glucose consumption and glutaminolysis. Thus, a ketogenic diet can selectively intensify metabolic oxidative stress, which has the potential to enhance the responsiveness of cancer cells to radiation and platinum chemotherapy without leading to increased toxicity in adjacent normal tissues [167]. Furthermore, since glucose is integral to the synthesis of growth-related metabolites through the pentose phosphate and glycolysis pathways, inhibiting glucose and glutamine can target both the glutamine and glutamine degradation as well as reduce acidity in the tumor microenvironment, thus inhibiting cancer cell proliferation.

Nutritional deficiencies and lack of fruits and vegetables are also associated with a higher OSCC risk. Several studies conducted worldwide have reported that a diet rich in fruits, vegetables, and lean protein correlates with a reduced risk of developing HNSCC, including OSCC [169–176]. Fruits and vegetables contain several bioactive compounds, including phytochemicals (carotenoids, flavonoids, and phenolics), micronutrients (vitamins and minerals), as well as fiber, which can play a protective role against oxidative stress and DNA repair [177, 178]. Phytochemicals are known to regulate several signaling pathways involved in glucose metabolism, gene expression, miRNAs, and epigenetic modifications such as Akt, MAPK, NF-κB, Wnt, and Notch [177, 179]. The NIH-AARP cohort [180] and EPIC study [181] reported a significant inverse association of OSCC with the consumption of total fruits and vegetables. Leoncini and colleagues [182] performed a pooled analysis and reported a reduced risk of developing oral/pharyngeal cancer by 39% in individuals with higher consumption of carotenoids as compared to those with lower consumption. Likewise, several studies have reported an inverse correlation between the intake of carotenoids (α-carotene, β-carotene, β-cryptoxanthin, lycopene, and lutein) and risk of HNSCC, including the subtypes [183–187]. Like carotenoids, other dietary antioxidants (vitamins and total flavonoids) are associated with a protective role against HNSCC [187–190]. Notably, polyunsaturated fatty acids like Omega-3 present in fish have shown a protective role against OSCC [176, 191, 192]. Lycopene also serves as a protective factor against oral cancer by modulating lipid peroxidation and reducing glutathione (GSH) levels [193]. Excessive iron intake is a significant dietary risk factor in the development of OSCC, as iron plays a crucial role in fundamental cellular processes like metabolism, cell growth, and proliferation [194]. High intake of iron is associated with the generation of nitrogen compounds and free radicals, contributing to cellular damage [194].

On the other hand, while meat is a source of proteins, minerals, and micronutrients, it consists of metabolites like nitrates and nitrites, which form *N*-nitroso compounds that underpin the onset of oral cancer [195, 196]. In addition, when cooked, red meat produces structurally related classes of carcinogens such as polycyclic aromatic hydrocarbons, N-nitroso compounds, aromatic amines, and heterocyclic aromatic amines [197]. Individuals consuming processed meat three or more times per week exhibited a significantly higher risk of HNSCC compared to those who did not include processed foods in their diets [170, 173]. A large prospective study reported an association between processed meat and HNC; however, the study did not report an association between red meat and HNC [198]. In addition, the study further reported a positive correlation between processed meat and OSCC [198]. The EPIC study [199] also reported a positive correlation between processed meat and cancer of the upper aerodigestive tract. In addition, a meta-analysis demonstrated a positive correlation between intake of processed meat and oral cavity and oropharyngeal cancer [200]. On the contrary, the NIH-AARP study [201] did not report an association between processed meat and OSCC. As the data obtained from the studies are contradictory [173, 198–201], further research is needed to correlate the association between intake of processed/red meat and an increased risk of OSCC as well as to understand their underlying mechanisms.

#### Obesity

Obesity is a metabolic disorder characterized by the accumulation of adipose tissue, elevated lipid levels, and insulin resistance [202]. A positive association was found between body-mass index (BMI) and HNSCC, especially in the cancers of the oral cavity, oropharynx, and hypopharynx in current smokers [203]. On the other hand, a pooled analysis of 20 cohort studies reported a larger BMI with an increased risk of HNSCC amongst nonsmokers [204]. In obese individuals, dysregulation of obesity-related genes and lipid signaling impairs disturbances in lipid metabolism, ultimately promoting tumor proliferation and progression [57, 205]. A retrospective study identified the upregulation of lipid metabolism-related genes (TGFB1, SPP1, and SERPINE1) in OSCC, which held prognostic significance [57]. To further elucidate the role of obesity in OSCC carcinogenesis, Peng et al. (2021) [206] used a 4NQO-induced mouse model exposed to a high-fat diet and normal fat diet and showed high-fat diet (HFD)induced obesity to significantly enhance the onset and progression of OSCC. Obesity-induced OSCC can be explained by lipid uptake-induced metabolic reprogramming [207], increased insulin levels [208], an increase in oxidative stress [209], as well as inflammation and alterations in the tumor microenvironment [210]. The authors additionally reported that HFD promotes an immunosuppressive microenvironment by functionally augmenting myeloid-derived suppressor cells (MDSCs) through elevated intracellular fatty acid uptake [206]. According to Zhang et al. (2022) [211], gene set enrichment analysis revealed a significant enrichment of genes associated with lipid metabolism in metastatic OSCC as compared to non-metastatic OSCC, thus suggesting a role of lipid metabolism in the metastatic process of OSCC. Furthermore, lipid metabolism showed a significant and positive correlation with epithelial-mesenchymal transition, PI3K/Akt/mTOR signaling, mTOR complex 1 (mTORC1) signaling, MYC targets V1, reactive oxygen species (ROS) pathway, and xenobiotic metabolism [211]. The study further identified three metastasis-associated events-related lipid metabolism-related genes (ACAT1, OXSM, and VAPA), indicating their role as potential therapeutic targets for OSCC [211].

Fatty acids, a major component of lipids, are involved in tumor cell proliferation, invasion, and metastasis [212]. Fan et al. (2022) [213] performed an analysis of The Cancer Genome Atlas (TCGA) data and microarray test and developed a risk-predictive scoring model comprising five genes (ACACB, FABP3, PDK4, PPARG, and PLIN5) related to fatty acid metabolism.

The above studies indicate that embracing a healthy lifestyle will be a highly effective approach to reducing the risk of cancer, and it includes regular exercise, maintaining a nutritious diet, abstaining from smoking, and moderating alcohol consumption.

### Genetic factors

OSCC is a heterogeneous malignancy and is characterized by dominant or recessive genetic alterations in various genes [214]. Proto-oncogenes and specific tumor suppressor genes (TSGs) are predominantly implicated in dominant changes, whereas recessive changes affect growth-inhibitory pathway genes or common TSGs, entailing gain and loss of function, respectively [215].

TP53, also known as the tumor protein 53, is a vital TSG significantly involved in maintaining cellular homeostasis and vital cellular processes, including cell cycle control, apoptosis, DNA repair, and metabolism. Approximately 40–70% of oral cancers harbor mutations in the TP53 gene, resulting in a non-functional product. Mutations in TP53 can be categorized as disruptive or non-disruptive [216]. While disruptive mutations entail abnormalities in the DNA binding domains, resulting in a substantial loss of function, non-disruptive mutations partially impact the normal functionality of TP53 [217]. Gain of function mutations in TP53 promote genomic instability, stimulate proliferation, invasion, and migration, and dysregulate metabolism, collectively contributing to therapeutic resistance and poor prognosis in OSCC [218].

FAT atypical cadherin 1 (FAT1) is another gene involved in OSCC, encoding a cadherin-like protein that participates in modulating cell adhesion, polarity, and migration. Additionally, FAT1 influences metabolic pathways such as glycolysis, glutaminolysis, and fatty acid synthesis. Recent studies have shown a critical role for the FAT1-YAP1 axis in regulating EMT [219] as well as for the maintenance of an active chromatin state [220] in HNSCC. Loss of the FAT1 gene induces a hybrid EMT state, where cells demonstrate both epithelial and mesenchymal characteristics [219]. This hybrid state correlates with enhanced tumor stemness, a characteristic feature that endows cancer cells with increased self-renewal abilities and resistance to conventional therapies [219]. Concurrently, FAT1 deletion activates YAP1, a key transcriptional co-activator that promotes oncogenic processes; the YAP1 signaling pathway triggers cell proliferation, survival, and migration [219]. The FAT1-YAP1 axis thus drives a more aggressive tumor phenotype, thus promoting metastasis by allowing tumor cells to detach from the primary site, invade the surrounding tissues, and establish secondary tumors at distant sites [221]. Furthermore, YAP1 maintains an active chromatin state by cooperating with BRD4, a bromodomain-containing protein that recognizes acetylated histones and regulates gene transcription [220]. The active chromatin state facilitated by the YAP1-BRD4 interaction results in the transcription of genes, either upregulation of oncogenes or suppression of tumor suppressor genes, to create a permissive environment for tumor growth and progression [220]. In OSCC as well, FAT1 is frequently mutated or downregulated, impairing its tumor-suppressing abilities. By acting as a tumor suppressor, FAT1 helps prevent the uncontrolled growth and spread of cancer cells. Its dysregulation in OSCC further emphasizes its significance in the development and progression of this type of oral cancer [222]. Thus, understanding the dual impact of the FAT1-YAP1 axis underlying EMT and chromatin dynamics aids in understanding its mechanism in cancer biology and underscores the therapeutic potential of targeting this pathway to disrupt the epigenomic and phenotypic support for tumor progression.

The phosphatidylinositol-4,5-bisphosphate 3-kinase catalytic subunit alpha (PIK3CA) gene encodes a subunit of phosphatidylinositol 3-kinase (PI3K), which is involved in multiple cellular processes essential for normal cell function. These processes include growth, proliferation, differentiation, motility, survival, and intracellular trafficking. Moreover, PIK3CA also plays a critical role in the insulin signaling pathway, which regulates glucose metabolism. However, in OSCC, PIK3CA is frequently mutated or overexpressed, leading to dysregulated cellular processes and aberrant glucose metabolism. These alterations are associated with a poor prognosis in OSCC patients, highlighting the impact of PIK3CA on disease progression [223, 224].

In addition, studies have demonstrated that PER1 and PER2 act as suppressors in OSCC by impeding glycolysis-induced cell proliferation *via* the PI3K/AKT pathway [225, 226]. In contrast, the mitochondrial serine hydroxymethyltransferase (SHMT2) is upregulated in OSCC and contributes to OSCC pathogenesis by promoting carbon metabolism by facilitating the conversion of serine to glycine [227]. Moreover, adenosine deaminase (ADA) levels in the saliva and serum were overexpressed in OSCC cases as compared to healthy subjects, indicating its potential role as a prognostic and diagnostic biomarker for OSCC [228, 229]. Likewise, GOT1, a primary player in amino acid metabolism, the urea cycle, and the TCA cycle was overexpressed in OSCC and was associated with tumor invasion and shorter survival [230, 231]. A recent study by Zhang and colleagues [232] evaluated clinical data and gene expression levels for OSCC cases from TCGA and identified differentially expressed metabolism-related genes (MRGs) associated with OSCC through differential analysis. The study identified 12 MRGs significantly linked to prognosis using univariate Cox analysis, and an OSCC clinical prognosis model based on 11 MRGs (SHMT2, HPRT1, POLD2, HADHB, POLE3, ADK, GOT1, ATIC, MGST1, ADA and GNPDA1) was constructed through Lasso-Cox analysis [232]. Furthermore, validation was performed using the Gene Expression Omnibus (GEO) database (GSE41613) as the validation set, thus indicating the use of MRGs in predicting OSCC survival [232]. On the other hand, another study validated the metastatic specificity of metastasis-associated enhancers (MAEs) in relation to three lipid MRGs (ACAT1, OXSM, and VAPA) and confirmed the regulation of these genes by a key transcription factor (CBFB) [211]. Notably, their findings demonstrated that the knockdown of CBFB resulted in the inhibition of proliferation, invasion, and lipid synthesis in OSCC cells [211]. Additionally, a recent study by Fan et al., (2022) [213] utilized microarray data, information TCGA, and quantitative real-time polymerase chain reaction (qRT-PCR) to identify differentially expressed genes associated with fatty acid metabolism in OSCC. Subsequently, they constructed a risk predictive scoring model using five fatty acid metabolism-related genes (ACACB, FABP3, PDK4, PPARG, and PLIN5) and assessed its performance through time-dependent receiver operating characteristic curve (ROC) analysis and revealed a significant association between the risk score derived from the five-gene signature and poor overall survival and disease-free survival in OSCC patients [213]. Furthermore, they demonstrated that combining the risk score with classic factors in a nomogram improved the predictive performance of the prognostic model (69). Another study by Pedro and colleagues [233] evaluated the expression of 84 genes related to oxidative stress in OSCC and non-tumor tissues by qRT-PCR and found that 21 genes encoding enzymes involved in antioxidant metabolism were differentially expressed in OSCC, with four genes (ATOX1, PRDX4, PRNP, and SOD2) being up-regulated and 17 genes (ALOX12, CAT, CSDE1, DHCR24, DUOX1, DUOX2, EPHX2, GLRX2, GPX3, GSR, GSTZ1, MGST3, PRDX1, OXR1, OXSR1, SOD1, and SOD3) being down-regulated.

### Epigenetic factors

Epigenetic changes are influenced by various environmental (diet, inflammation or viral infections) and genetic factors, impacting the expression and function of genes involved in diverse cellular processes like cell growth, differentiation, and drug resistance [234]. Epigenetic alterations are a hallmark feature of human cancers and are increasingly recognized to work in conjunction with metabolism to promote the development and progression of cancer [235]. Modifications, such as DNA methylation, histone modification, and various RNA-mediated mechanisms, have an impact on gene expression during transcription [236]. Oncogene-induced metabolic reprogramming influences the epigenetic landscape by modulating the activity of enzymes involved in DNA and histone modifications [236]. Conversely, epigenetic mechanisms regulate the expression of metabolic genes, resulting in alterations to the cellular metabolome [236]. By altering the structure and function of chromatin, these epigenetic changes can profoundly impact gene expression, leading to the dysregulation of key pathways involved in cell growth, differentiation, and survival. This bidirectional relationship underscores the close connection between epigenetic modifications and metabolic changes in the context of cancer. Thus, a better understanding of the interplay between epigenetic and genetic alterations in cancer could lead to new strategies for the prevention and treatment of this disease [237]. Over the last decade, several single-cell and single-nucleus next-generation sequencing techniques have been developed, metamorphosing the detection of rare cell populations and extending our understanding of epigenetic regulation underpinning tumor heterogeneity at the individual cell levels [238–241]. These advanced techniques extend beyond transcriptional transcriptomics of individual cells to include several epigenetic approaches, such as the single-nucleus Assay for Transposase Accessible Chromatin (snATAC-seq), which allows chromatin accessibility [238]. SnATAC-seq identifies regions with low nucleosome occupancy (open chromatin) which are vital for efficient transcription factor binding and reveals cellular heterogeneity even in those populations that are transcriptionally or morphologically homogeneous [238]. On the other hand, single-cell sequencing allows comprehensive analysis of different aspects of tumor heterogeneity [239, 241]. It helps to identify novel tumor cell subtypes and their epigenetic modifications, study cellular crosstalk *via* ligand-receptor expressions, and map the differentiation paths of cancer stem cells [241]. In addition, single-cell sequencing can track rare mutations triggering metastasis and drug resistance as well as provide spatial and temporal gene expression data, revealing the intricate relationship between cell distribution and gene regulation within tumors [241]. However, though single-cell multi-omics analysis holds great promise for studying tumor heterogeneity, it is still in its primary stages and faces substantial technical and computational hurdles. A few of the known limitations include acquiring accurate data requiring precise matching between omics, potential errors and biases, high costs, and low throughput, limiting its clinical use [242]. These challenges can compromise our data analysis and impede our understanding of tumor heterogeneity [242]. Nonetheless, integrating spatial omics with single-cell omics (spatial proteomics, transcriptomics, and spatial epigenomics) has enhanced our comprehension of cancer evolution in both temporal and spatial contexts; however, the field of spatial omics in cancer research remains nascent, and further work is essential to convert spatial information into DNA barcodes or utilize imaging-based techniques to uphold the spatial framework of each cell [243]. In OSCC, the main epigenetic mechanisms involved are DNA methylation, histone modifications, non-coding RNAs, and chromatin remodeling [244].

DNA methylation was found to be altered in OSCC, with global hypomethylation and gene-specific hypermethylation observed. These changes affect genes responsible for critical cellular processes such as cell cycle regulation, DNA repair, apoptosis, and metastasis [245]. On the other hand, histone modifications, such as acetylation, methylation, and phosphorylation, play a crucial role in regulating gene expression and have significant implications for the development and progression of OSCC. Histone acetylation is associated with active gene expression, while histone methylation and phosphorylation can have activating or repressive effects depending on the location of such modification [245]. Epigenome-wide DNA methylation profiling of 87 Gingivo-buccal oral squamous cell carcinoma (OSCC-GB) patients reported upregulation of PD-L1 and CD80 due to significant promoter hypomethylation, thus inhibiting apoptosis of OSCC-GB cells due to the immunosuppressive characteristics of PD-L1/PD-1 interactions [246]. The study also reported epigenetic modifications to induce the expression of DNA methyltransferase (DNMT)-3B and inhibit TET1 expression [246]. DNMT3B serves as a de novo DNA methyltransferase, facilitating the transfer of a methyl group from S-adenosylmethionine to the C-5 position of cytosine to generate 5-methylcytosine (5mC) without the need for a template [247]. Previous studies have also reported elevated levels of DNMT3B to epigenetically inactivate TSGs, contributing to the development of oral tumors [248, 249]. On the other hand, promoter CpG methylation-induced loss of TET1 expression has been previously linked to head and neck cancer [250]. Pathway analysis in OSCC-GB reported enhanced arachidonic acid metabolism, PPAR signaling, and B cell receptor signaling pathways [246]. Moreover, the cluster of Krüppel-type zinc finger protein genes, such as ZNF132 and ZSCAN18 on chromosome 19q13, is epigenetically silenced in OSCC [251]. In addition, while epigenetic silencing of CDON, CPEB1, GAS7, ID4, NUPR1, OSR1, SELENBP1, TGFBR3, ZSCAN18, and ZBTB16 has been reported in OSCC [246, 252–255], epigenetic upregulation of LCK, SULF1, SEMA3C, and TRPM2 was observed in patients with OSCC or oral lichen planus [246, 256, 257].

miRNAs, small RNA molecules, regulate gene expression by binding to target messenger RNAs (mRNAs) and inhibiting their translation or promoting their degradation. Dysregulated miRNAs in OSCC also can impact crucial cellular processes such as cell proliferation, apoptosis, and metastasis [245]. Ping Xu and colleagues [258] reported that miRNA-340 is downregulated in OSCC and regulates the metabolic shift in OSCC by targeting Glut1, a key protein for glucose uptake and glycolysis. The authors reported that inhibition of miRNA-340 in OSCC cells increased Glut-1 expression, lactate secretion, glucose uptake rate, cell proliferation, and colony formation [258]. Similarly, miRNA-143 was downregulated in OSCC tissues and cell lines and was found to promote OSCC cell growth, invasion, and glucose metabolism by directly targeting HK2, a key enzyme in glycolysis [36].

Epigenetic factors encompass proteins or non-coding RNAs that induce modifications to DNA’s structure or functionality without modifying its sequence. These factors possess the ability to control the activation or suppression of genes through alterations in DNA methylation, histone modification, or RNA interference. Long non-coding RNAs (lncRNAs) exert their influence on glucose metabolism in cancer cells through diverse mechanisms. The lncRNA H19/miR-675-5p/PFKFB3 signaling pathway plays a role in promoting oral cancer development by altering glycolysis in cancer-associated fibroblasts [259]. Epigenetic alterations can serve as useful biomarkers for early detection, risk assessment, prognosis, and prediction of response to therapy in oral diseases. Epigenetic drugs, such as DNA methyltransferase inhibitors, histone deacetylase inhibitors, and non-coding RNA mimics or inhibitors, can modulate the epigenetic landscape and restore normal gene expression in oral tissues [260]. Interestingly, drugs such as dasatinib, calcitriol, tamoxifen, and aspirin were found to induce the reversal of gene expressions related to CD274, CD80, DNMT3B, TET1, PPARG, and PIK3CD [246].

Some of the genes that are dysregulated by epigenetic alteration in OSCC are listed in Table 1.

**Table 1:** Epigenetically altered genes in OSCC Metabolism

Genes	Related pathway	Related metabolism pathway	Epigenetic Alteration	Responsible epigenetic factor
DNMT3B	DNA methylation	Methionine cycle	Overexpression	Mutations or copy number variations
TET1	DNA demethylation	Folate cycle	Downregulation	Mutations or copy number variations
CDKN2A	Cell cycle	Purine metabolism	Promoter hypermethylation	DNMT
RASSF1A	Apoptosis	Glutathione metabolism	Promoter hypermethylation	DNMT
DAPK	Apoptosis	Glycolysis	Promoter hypermethylation	DNMT
TIMP3	Invasion	Glycosaminoglycan degradation	Promoter hypermethylation	DNMT s
MYC	Cell survival	Glycolysis and glutaminolysis	Promoter hypomethylation	DNMT or histone acetyltransferases

## Technical aspects of metabolomics diagnostic strategies workflow

**Fig. 2:**
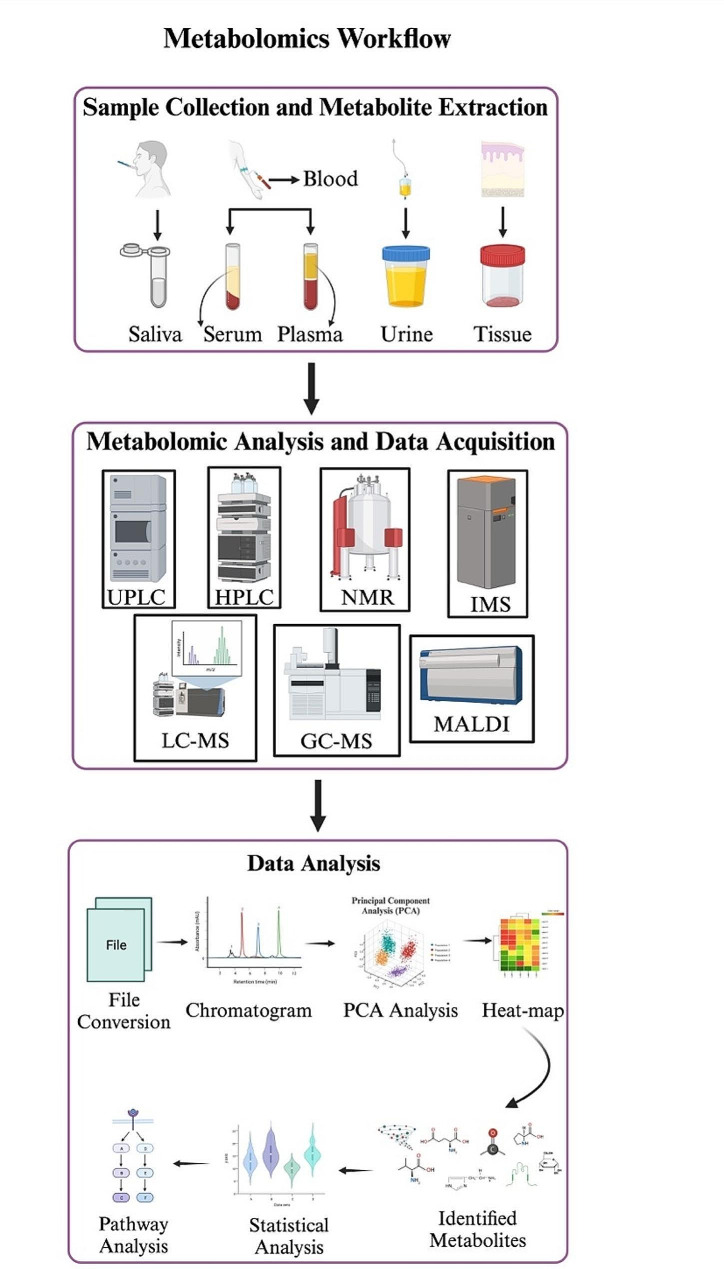
Overview of the metabolomics workflow. This comprehensive schematic outlines the step-by-step process involved in conducting metabolomics studies. The initial step of the metabolomics workflow includes sample collection from tissues, biofluids, or cells; the workflow proceeds through meticulous sample preparation, including extraction and derivatization. The prepared samples undergo analysis using state-of-the-art techniques, such as liquid chromatography-mass spectrometry (LC-MS), gas chromatography-mass spectrometry (GC-MS), ultraperformance liquid chromatography (UPLC), high-performance liquid chromatography (HPLC), nuclear magnetic resonance (NMR), imaging mass spectrometry (IMS) or matrix-associated laser desorption ionization (MALDI) which enable high-resolution detection and quantification of metabolites. The subsequent phase encompasses data processing, involving metabolite identification, quantification, and normalization. Statistical analyses are then applied to discern significant metabolic alterations. This systematic metabolomics workflow serves as a powerful tool for unraveling the intricate metabolic signatures, thereby contributing crucial insights to the understanding of its development and progression. This figure is created with BioRender.com

The metabolomics workflow consists of several steps, as exemplified in Fig. 2. The initial phase of planning a metabolomics study entails meticulous consideration of sample collection, storage, and preparation (Fig. 2). Biological fluids (blood serum or plasma, urine, saliva) and tissues (oral cavity, lip, and tongue) are the samples commonly used for OSCC metabolomic studies. The advantage of using fluids is the easy and non-invasive sample collection. Besides, fluid samples can be used for nuclear magnetic resonance (NMR) or mass spectrometry (MS) measurements without or with minimal treatment [261]. The main challenge with blood metabolomics is that blood metabolite composition reflects the current biochemical state of the whole body. Hence, it is often difficult to separate the metabolomic changes caused by a particular disease (OSCC) from changes induced by other factors (other pathologies, genetic variations, age, gender, diet, lifestyle, and so on). The same is true for urine metabolomics. Therefore, saliva is the fluid most suitable for OSCC-related metabolomics: it is in direct contact with the affected tissues, and the influence of other diseases is not as significant as in the case of blood and urine [261].

Fluid metabolomics identifies compounds whose concentrations in the blood or saliva change significantly, either due to enhanced excretion from cells or the increased uptake by cells. On the other hand, tissue metabolomics reflects the metabolic processes directly in cells, and changes in intracellular metabolome under disease development can be much greater than those in surrounding fluids [262]. Hence, the metabolomic analysis of tissues is considered the most informative both for diagnostic purposes and for a better understanding of the biochemical processes occurring during the OSCC development [262]. However, the major disadvantages of tissue metabolomics are the invasiveness of sample collection and more complex pre-analytical sample preparation [263].

Sample preparation is the most crucial and important part of metabolomic analysis. It has been estimated that the pre-analytical phase accounts for up to 80% of all testing errors in clinical practice [264]. The tissue sample preparation includes sample homogenization, quenching metabolic activity, protein precipitation, and extraction of low molecular weight compounds as a separate fraction [265–267]. This is usually performed with the use of organic solvents such as acetonitrile, chloroform, methanol, and ethanol [267–272]. The metabolomic extracts often need to be concentrated to increase the detection sensitivity and to replace the solvent with a more suitable one for the analysis. The most common laboratory techniques for solvent evaporation are rotary vacuum evaporation and lyophilization. In metabolomic studies of fluids, some of the sample preparation steps (for example, sample homogenization) can be omitted, but quenching metabolic activity and protein precipitation are often still necessary. The major sources of errors at the pre-analytical stage are incomplete metabolite extraction, chemical degradation of metabolites during the sample preparation, and metabolite transformation due to enzymatic reactions [273–278]. Depending on the method of metabolomic analysis, the sample preparation protocol may significantly vary. For instance, gas chromatography often requires metabolite derivatization, while magic-angle spinning NMR (MAS-NMR) does not require sample preparation at all [279].

The next step involves analyzing biofluid or tissue samples obtained from both healthy individuals and cancer patients using mass spectrometry (MS) and nuclear magnetic resonance (NMR) spectroscopy (Fig. 2). NMR spectroscopy allows for the fast and non-destructive analysis of complex metabolite mixtures with notable reproducibility (coefficient of variation ~ 1–2%) [25]. By leveraging unique chemical shifts and multiple patterns in NMR spectra, specific metabolites can be identified and quantified, enabling the detection and characterization of numerous metabolites in a single experiment [280]. The main advantage of NMR-based metabolomics is that the signal intensities in ^1^H NMR spectra are proportional to the metabolite concentrations and do not depend on metabolite properties, thus simplifying metabolite quantification [280]. For this reason, NMR is likely the most suitable tool for quantitative metabolomics, especially when the absolute values of metabolite concentrations in a tissue (in units of nmol per gram) are measured [280]. The disadvantage of NMR is its relatively low sensitivity: typically, about 50–100 metabolites can be reliably identified and quantified from an NMR spectrum of the metabolomic extract [280]. On the other hand, MS detection offers inherent high sensitivity, typically at the picogram level, making it a valuable method for measuring metabolites in complex biological fluids [281]. In numerous metabolomics studies, various MS techniques coupled with separation methods like gas chromatography (GC), capillary electrophoresis (CE), and liquid chromatography (LC), along with their respective variants, have been extensively employed [282–284]. These techniques allow the detection of thousands of metabolomic features in a single experimental run, as well as the identification of hundreds of compounds in a biological fluid or metabolomic extract [285, 286]. The disadvantage of MS-based metabolomics is that the intensities of MS signals depend on the ionization coefficient specific to each compound. For metabolite quantification, one needs to construct a calibration curve for every compound under study, which is rather labor consuming. Besides, the intensities of MS signals might be influenced by the ion suppression effect [287]. Due to these limitations, the majority of MS-based metabolomic studies are performed at a semi-quantitative level, where rather than absolute values of metabolite concentrations, ratios of metabolite abundances between experimental and control groups are determined. However, as compared to MS, NMR offers distinct advantages in providing quantitative measurements of metabolite concentrations, facilitating more precise and reliable analysis in metabolomics research.

After collecting raw metabolomic data with quality control measures, the next step involves bioinformatics and data analysis (Fig. 2). Multivariate statistical analyses are commonly employed to simplify and interpret such complex data [288–290]. The data from these techniques are analyzed using advanced chemometric methods, which enable a powerful platform for translational research, clinical applications, and diagnostics [289, 290]. These analyses play a crucial role in reducing the dimensionality of the data and, more importantly, in distinguishing between “disease” and “control” populations by identifying differences in the signals of multiple metabolites in different samples [291]. The most popular platform for chemometric data analysis is the MetaboAnalyst web platform (www.metaboanalyst.ca) [292], which contains virtually all tools needed for comprehensive metabolomics data analysis, interpretation, and integration with other omics data.

Usually, the first step in the data analysis is the comparison of data sets obtained for samples from “disease” and “control” groups. Since metabolomic data contain thousands of numbers, it is impossible to carry out the comparison manually. Principal Component Analysis (PCA) and Partial Least Squares-Discriminant Analysis (PLS-DA) are the commonly used methods of data dimensionality reduction in metabolomics [293]. The main goal of applying these methods to experimental data sets is to establish the presence or absence of differences between the “disease” and “control” groups. If a difference is detected, the next step is the identification of metabolites responsible for the difference [293]. Heat-maps help visualize metabolites with reduced or enhanced abundances (differential metabolites), and Volcano plots show metabolites whose concentrations demonstrate a statistically significant difference between the “disease” and “control” groups [293]. The final step is the determination of metabolic pathways most affected by the disease development. Metabolite Set Enrichment Analysis (MSEA) compares detected differential metabolites with metabolite sets belonging to different metabolic pathways and identifies the most affected pathways considering the number of hits, fold change, and statistical significance of the change [293]. The frequently used tools for MSEA metabolite set libraries include the SMPDB [294] and KEGG [295] databases. Additionally, the construction of receiver operating characteristic (ROC) curves is a tool often used for the determination of potential metabolomic biomarkers for the disease under study [296].

## Clinical applications of metabolomics in oral squamous cell carcinoma

Metabolomics can capture the alterations in the metabolic processes and networks that occur during cancer initiation and progression. By analyzing the metabolite profiles of cancer patients, precancerous patients, and healthy controls, researchers can discover potential biomarkers that can differentiate between various stages and types of cancers. These biomarkers can also shed light on the underlying mechanisms and pathways of cancer development and tumor growth. Several studies have applied metabolomics to investigate the metabolic alterations in OSCC and to identify potential biomarkers for diagnosis and prognosis. Various biological fluids, including saliva, blood, serum, plasma, and urine, have been employed in metabolomic-based investigations [297–300]. These biofluids harbor hundreds to thousands of detectable metabolites, offering non- or minimally invasive sampling options [301]. Furthermore, cell and tissue extracts serve as additional sample sources for metabolomic studies [301].

Table 2 summarizes the different techniques used in OSCC metabolomic studies to identify the metabolites in biological fluids and tissue samples.

**Table 2:** Metabolomic techniques used to identify metabolites in OSCC in different clinical samples

Saliva Samples		
Technique	Identified Differential Metabolites	Reference
Capillary Electrophoresis Mass Spectrometry (CE-MS)	Choline, p-hydroxyphenylacetic acid, 2-hydroxy-4-methylvaleric acid, valine, 3-phenyllactic acid, leucine, hexanoic acid, octanoic acid, terephthalic acid, γ-butyrobetaine, 3-(4-hydroxyphenyl) propionic acid, isoleucine, tryptophan, 3-phenylpropionic acid, 2-hydroxyvaleric acid, butyric acid, cadaverine, 2-oxoisovaleric acid, N6,N6,N6-trimethyllysine, taurine, glycolic acid, 3-hydroxybutyric acid, heptanoic acid, alanine, urea	[315]
Capillary Electrophoresis Time-Of-Flight Mass Spectrometry (CE-TOF-MS)	Pyrroline hydroxycarboxylic acid, leucine plus isoleucine, choline, tryptophan, valine, threonine, histidine, pipecolic acid, glutamic acid, carnitine, alanine, piperidine, taurine, alpha-aminobutyric acid, phenylalanine, betaine, serine, tyrosine, glutamine, beta-alanine, cadaverine	[303]
	3PG, Pipecolate, Spermidine, Met, SAM, 2AB, Tryptophan, Valine, Hypoxanthine, di-glycine, Trimethylamine N-oxide, Guanine, Guanosine, Taurine, Choline, Cadaverine, Threonine	[317]
High-performance liquid chromatography (HPLC)	Glutathione	[305]
Ultraperformance Liquid Chromatography (UPLC)	Choline, betaine, pipecolinic acid, and L-carnitine	[316]
UPLC coupled with quadrupole/time-of-flight mass spectrometry (UPLC-QTOF-MS)	γ-aminobutyric acid, phenylalanine, valine, n-eicosanoic acid, and lactic acid	[310]
Gas Chromatography-Mass Spectrometry (GC-MS)	Decanedioic acid, 2-methyloctacosane, eicosane, octane, 3,5-dimethyl, pentadecane, hentriacontane, 5, 5-diethylpentadecane, nonadecane, oxalic acid, 6-phenylundecanea, l-proline, 2-furancarboxamide, 2-isopropyl-5-methyl-1-heptanol, pentanoic acid, Docosane	[311]
	2-ketoglutaric acid, 2-hydroxyglutaric acid, 3-hydroxypropionic acid, 4-hydroxyphenylatic acid, galacturonic acid, gluconic acid, hippuric acid, indol-3-acetic acid, isocitric acid, malic acid, pantothenic acid, protocatechuic acid, ureidosuccinic acid, spermidine, dihydroxyacetone phosphate, inosine, lactitol, lyxose, maltose, methionine, O-phospho-serine, ribose 5-phosphate, sorbose, thymidine, and uracil	[312]
Conductive Polymer Spray Ionization Mass Spectrometry (CPSI-MS)	Putrescine, cadaverine, thymidine, adenosine, 5-aminopentoate, hippuric acid, phosphocholine, glucose, serine, adrenic acid	[313]
RPLC and Hydrophilic Interaction Chromatography combined with TOF-MS	Lactic acid, Hydroxyphenyllactic acid, N-nonanoylglycine, 5-hydroxymethyluracil, Succinic acid, Ornithine, Hexanoylcarnitine, Propionylcholine, Carnitine, 4-hydroxy-L-glutamic acid, Acetylphenylalanine, Sphinganine, Phytosphingosine	[314]
**Serum Samples**		
Nuclear Magnetic Resonance Spectroscopy (NMRS)	Valine, ethanol, lactate, alanine, acetate, citrate, phenylalanine, tyrosine, methanol, formaldehyde, formic acid, glucose, pyruvate, acetone, acetoacetate, 3-hydroxybutyrate and 2-hydroxybutyrate, choline, betaine, dimethylglycine, sarcosine, asparagine, and ornithine	[319]
GC-MS	Glucose, ribulose, methionine, and ketoisoleucine	[321]
CPSI-MS	Sphingosine 1-phosphate, PC(34:6), DG(34:4), oleamide, lysoPA(18:0),c glucuronide, taurocholic acid, palmitic amide, palmitoleic acid, pyridinoxamine, adenine, kynuramine, cytosine, glucose, lysoPC(18:3), PS(14:1/16:1), creatinine, proline, betaine, MG(18:2/0:0/0:0), 2-Hydroxyadipic acid, glycerophosphocholine, lysoPC(16:0), lysoPC(20:3), DG(16:0/24:0), 2-Stearoylglycerophosphoglycerol, 3-Hydroxy-N6,N6,N6-trimethyl-lysine, PC(P-40:6), hydroxycholesterol, N-methylnicotinamide, propionylcholine, lysoPC(20:4), lysoPC(18:1), lysoPE(20:3), 2-Piperidinone, lysoSM(d18:1), erythritol, nonanoylcarnitine, 6-keto-decanoylcarnitine, PG(34:0)	[322]
(1)H and (13)C NMR	Choline, trimethylamine N-oxide, malonic acid, lactate, D-glucose	[323]
QTOF-MS	Estradiol-17-beta-3-sulfate, L-carnitine, 5-methylthioadenosine, 8-hydroxyadenine, 2-methylcitric acid, putrescine, estrone-3-sulfate, 5,6-dihydrouridine and 4-hydroxypenbutolol glucuronide	[324]
**Plasma Samples**		
NMR-MS	Acetate, acetoacetate, acetone, alanine, creatine, formate, proline, sarcosine, threonine, tyrosine	[325]
1 H NMR	Valine, threonine, glutamine, creatinine, propionate, acetone, acetate, and choline	[320]
UHPLC quadrupole-Orbitrap high-resolution accurate mass spectrometry (UHPLC/Q-Orbitrap HRMS)	Uric acid, glutamate, acetylcarnitine, leucine, phenylalanine, phenylacetylglutamine, 2,4-dihydroxyacetophenone, cysteine, acetyl phenylalanine, 4-Nitrophenol, indoxyl sulphate, octanoylcarnitine, glycitin, decanoylcarnitine, monobutyl phthalate, and cholic acid	[326]
**Urine Samples**		
GC-MS	Alanine, leucine, cystine, valine, serine, cysteine, 6-Hydroxynicotic acid, Hippurate, phenylalanine, histamine, tyrosine, and tryptophan	[327]
**Tissue Samples**		
GC-MS	Glycerol, xylitol, 2-Aminoethanol, nicotinamide, oxalate, homoserine, uracil, putrescine, inosine, glycine, hypoxanthine, valine, aspartic acid, glutamic acid, proline, 3-hydroxyisovaleric acid, ascorbic acid, coniferyl aldehyde, malonic acid, pipecolic acid, S-Benzyl-L-cysteine, asparagine, sarcosine, threonine, phosphate, glyceraldehyde, pyroglutamic acid, acetoacetic acid, O-phosphoethanolamine, 2-Aminobutyric acid, glucose, tyrosine, trans-4-hydroxy-L-proline, alanine, phenylalanine, serine, methionine, mannitol and histidine	[321]
	Alanine, glutamic acid, glutamine, glycine, lysine, norleucine, proline, serine and threonine	[337]
Desorption Electrospray Ionization Mass Spectrometry Imaging (DESI-MS)	Oleamide, DG(34:4), PC(34:6), lysoSM(d18:1), erythritol, lysoPC(18:3), nonanoyl carnitine, lysoPC(20:4), lysoPE(20:3) and Sphingosine-1P	[322]
	Arachidonic acid, ecosatrienoic acid, eicosadienoic acid, eicosenoic acid, docosahexaenoic acid, docosapentaenoic acid, adrenic acid, docosatrienoic acid, erucic acid. tetracosatetraenoic acid, nervonic acid, hexacosenoic acid, and octadecanoic acid	[336]
High-resolution magic-angle spinning (HR-MAS) proton NMRS	Lactate, leucine, isoleucine, valine, alanine, glutamine, glutamate, aspartate, glycine, phenylalanine, tyrosine, creatine, taurine, glutathione, and triglycerides	[332]
	Glutamate, choline, phosphocholine, lactate, acetate, taurine, glycine, leucine, lysine, isoleucine, alanine, creatine and PUFA	[333]
UHPLC-tandem mass spectrometer (UHPLC -MS/MS)	Glutamine, ornithine, histidine, valine, lysine, glutamate, aspartic acid, proline, alanine, phenylalanine, and serine	[334]
Imaging Mass Spectrometry (IMS) along with MS/MS	Phosphatidylcholine (16:0/16:1) and Phosphatidylcholine (18:1/20:4)	[335]

**DG**: diacylglycerol; **lysoPA**: lysophosphatidic acid; **lysoPC**: lysophosphatidylcholine; **lysoPE**: lysophosphatidylethanolamine; **lysoSM**: lysosphingomyelin; **MG**: monoacylglycerol; **PC**: phosphatidylcholine; **PC(P-40:6)**: plasmenylcholine; **PG**: phosphoglycerate; **PS**: phosphatidylserine; **PUFA**: polyunsaturated fatty acids; **SAM**: S-adenosylmethionine

Table 3 shows the different metabolic pathways associated with the identified metabolites mentioned in Table 2 above.

**Table 3:** **Identified metabolities and their associated Metabolomic Pathways**

Metabolic Pathway	Metabolites
Amino-Acid Metabolism	2-aminobutyric acid [317, 321], 2-hydroxyvaleric acid [303], 2-methylcitric acid [324], 2,4-dihydroxyacetophenone [326], 3-(4-hydroxyphenyl) propionic acid [303, 312], 3-hydroxyisovaleric acid [321], 3-hydroxy-N6,N6,N6-trimethyl-lysine [322], 3-phenylpropionic acid [303], 4-hydroxy-L-glutamic acid [314], 4-hydroxyphenylatic acid [312], 5-methylthioadenosine [324], α-aminobutyric acid [303], β-alanine [303], γ-aminobutyric acid [310], acetylphenylalanine [314, 326], alanine [303, 315, 319, 321, 325, 327, 332, 333, 337], asparagine [319, 321], aspartic acid (aspartate) [321, 332, 334], betaine [303, 314, 319, 322], cadaverine [303, 315, 317], creatine [325, 332, 333], cystine [327], cysteine [326, 327], formate [320, 325], glutamine [303, 320, 332, 334, 337], glutamic acid (glutamate) [315, 321, 326, 332–334, 337], glutathione [305, 332], di-glycine [317], glycine [319, 321, 332, 333, 337], histidine [303, 321, 334], homoserine [321], hydroxyphenyllactic acid [314], isoleucine [303, 315, 332, 333], ketoisoleucine [321], kynuramine [322], L-proline [311], leucine [303, 317, 327, 333, 334, 337], lysine [333, 334, 337], methionine [312, 317, 319, 321], N6,N6,N6-trimethyllysine [315], norleucine [337], O-phospho-serine [312], oxalate [321], phenylacetylglutamine [326], phenylalanine [303, 310, 319, 321, 326, 327, 332, 334], pipecolic acid (5-aminopentoate, pipecolate) [303, 313, 317, 321], pipecolinic acid [314], proline [321, 322, 325, 334, 337], pyridinoxamine [322], pyroglutamic acid [321], SAM [317], sarcosine [319, 325], serine [303, 313, 321, 327, 334, 337], threonine [303, 317, 320, 321, 325, 337], trans-4-hydroxy-L-proline [321], tryptophan [303, 315, 317, 327], tyrosine [303, 319, 321, 325, 327, 332], valine [303, 305, 311, 317, 321, 322, 326, 333, 334, 337]
Carbohydrate Metabolism	Galacturonic acid [312], glyceraldehyde [321], lactate [319, 323, 332, 334], lactic acid [310, 314], lactitol [312], lyxose [312], maltose [312], mannitol [321], sorbose [312], xylitol [321]
Fatty Acid Metabolism	6-keto-decanoylcarnitine [322], acetate [319, 320, 325, 333], acetone [319, 320, 325], acetoacetate [319, 325], acetylcarnitine [326], adrenic acid [313, 336], arachidonic acid [336], butyric acid [303], carnitine [303, 314], DG(34:4) [322], decanedioic acid [311], decanoylcarnitine [326], docosahexaenoic acid [336], docosapentaenoic acid [336], docosatrienoic acid [336], eicosadienoic acid [336], eicosenoic acid [336], ecosatrienoic acid [336], erucic acid [336], hexacosenoic acid [336], hexanoylcarnitine [314], L-carnitine [314, 324], lysoPA(18:0) [322], lysoPC(16:0/18:1/18:3/20:3/20:4) [322], lysoPE(20:3) [322], n-eicosanoic acid [310], N-nonanoylglycine [314], nervonic acid [336], nonanoyl carnitine [322], octadecanoic acid [336], octanoic acid [303], octanoylcarnitine [326], oleamide [322], p-hydroxyphenylacetic acid [303], pantothenic acid [312], pentanoic acid [311], propionate [320], PUFA [333], taurine [303, 315, 317, 332, 333], tetracosatetraenoic acid [336], triglycerides [332]
Glucose Metabolism	3PG [317], alanine [303, 315, 319, 321, 325, 327, 332, 333, 337], D-glucose [323], dihydroxyacetone phosphate [312], gluconic acid [312], glucose [313, 319, 321, 322]
Ketogenesis	3-hydroxybutyrate and 2-hydroxybutyrate [319]
Lipid Metabolism	2-aminoethanol [321], 2-hydroxyadipic acid [322], 2-stearoylglycerophosphoglycerol [322], acetoacetic acid [321], cholic acid [326], choline [303, 305, 310, 317, 321, 324, 326, 334], DG(16:0/24:0) [322], glycerol [321], glycerophosphocholine [322], hydroxycholesterol [322], lysoSM(d18:1) [322], MG(18:2/0:0/0:0) [322], O-phosphoethanolamine [321], palmitic amide [322], palmitoleic acid [322], PC(34:6) [322], PC(P-40:6) [322], phosphocholine [313, 333], phosphatidylcholine [335], phytosphingosine [314], PG(34:0) [322], propionylcholine [314, 322], PS(14:1/16:1) [322], sphinganine [314], sphingosine-1P [322], taurocholic acid [322]
Nucleotide Metabolism	4-hydroxypenbutolol glucuronide [324], 5-hydroxymethyluracil [314], 5,6-dihydrouridine [324], 8-hydroxyadenine [324], adenine [322], adenosine [313], cytosine [322], hypoxanthine [317, 321], glucuronide [322], guanine [317], guanosine [317], inosine [312, 321], thymidine [312, 313], uracil [312, 321], ureidosuccinic acid [312], uric acid [326],
One-Carbon Metabolism	Acetone [319, 320, 325], dimethylglycine [319], formaldehyde [319], formate [319, 325], glycine [319, 321, 332, 333, 337], SAM [317], threonine [303, 317, 320, 321, 325, 337]
Ornithine Cycle	Ornithine [314, 319, 334], putrescine [313, 321, 324], spermidine [312, 317], urea [315]
Pentose Phosphate Pathway	Erythritol [322], ribose 5-phosphate [312], ribulose [321]
Tricarboxylic acid (TCA) cycle	2-hydroxyglutaric acid [312], 2-ketoglutaric acid [312], acetate [310, 317, 325, 327], citrate [319], decanedioic acid [311], isocitric acid [312], malic acid [312], malonic acid [321, 323], oxalic acid [311], pantothenic acid [312], pyruvate [319], succinic acid [314]
Xenobiotic Metabolism	4-Nitrophenol [326], hippurate [327], hippuric acid [312, 313], monobutyl phthalate [326], piperidine [303], S-benzyl-L-cysteine [321]
Other Metabolic Pathways	6-Hydroxynicotic acid [327], γ-butyrobetaine [303], cadaverine [313], estradiol-17-beta-3-sulfate [324], estrone-3-sulfate [324], ethanol [319], indoxyl sulphate [326], methanol [319],, N-methylnicotinamide [322], nicotinamide [321], piperidinone [322], protocatechuic acid [312], trimethylamine N-oxide [317, 323]

**DG**: diacylglycerol; **lysoPA**: lysophosphatidic acid; **lysoPC**: lysophosphatidylcholine; **lysoPE**: lysophosphatidylethanolamine; **lysoSM**: lysosphingomyelin; **MG**: monoacylglycerol; **PC**: phosphatidylcholine; **PC(P-40:6)**: plasmenylcholine; **PG**: phosphoglycerate; **PS**: phosphatidylserine; **PUFA**: polyunsaturated fatty acids; **SAM**: S-adenosylmethionine

### Biofluids

The challenge in promptly diagnosing OSCC stems from the difficulty in distinguishing it from other oral premalignant lesions using non-invasive or minimally invasive techniques that match the accuracy of histological diagnosis. Consequently, researchers have undertaken comparisons of metabolites in readily collectible fluids from individuals with OSCC or premalignant lesions and compared them to samples from healthy individuals to pinpoint distinctive metabolites associated with OSCC. This exploration aims to provide clinicians with valuable diagnostic insights. The following section will review the metabolomic-based investigations using biofluids (saliva, blood, serum, plasma, and urine) as well as tissue samples to study metabolomics in OSCC.

#### Saliva

Saliva serves as an advantageous diagnostic biofluid compared to other specimens, such as blood and urine, as it is non-invasive, easy to collect, rapid, cost-effective, and being in contact with tumor tissue of the OSCC carries a lot of cancer cell-released molecules and biomarkers [302].

Sugimoto et al. (2010) [303] identified 28 metabolites, of which salivary polyamine levels were significantly elevated in oral cancer samples as compared to control samples. Elevated polyamine levels have been linked to increased cell proliferation, reduced apoptosis, and heightened expression of genes influencing tumor invasion and metastasis [304], thus indicating a vital role of polyamine in OSCC. On the other hand, salivary glutathione was significantly elevated in oral and pharyngeal SCC samples as compared to healthy controls; however, due to inconsistency in the concentrations, glutathione is yet to be defined as a definitive diagnostic marker for SCC [305].

OSCC cells produce cytokines that can modulate the metabolic pathways and enzymes involved in glucose and lipid metabolism [306]. While some cytokines can enhance glucose and lipid metabolism by stimulating insulin secretion, enhancing glucose uptake, and activating fatty acid oxidation, other cytokines can impair glucose and lipid metabolism by inducing insulin resistance, promoting lipogenesis, and inhibiting fatty acid oxidation [306]. The dysregulation of cytokine-mediated inflammation and metabolism may contribute to the pathogenesis of oral diseases, such as periodontitis and OSCC. Salivary cytokines may reflect the metabolic status of oral cells and serve as potential biomarkers for diagnosis and prognosis [307, 308]. Zhang et al. (2021) [307] conducted a study to explore the use of salivary biomarkers for detecting periodontitis, a chronic inflammatory disease of the gums with a plausible relation to OSCC. The study reported notable efficacy of salivary levels of IL-1β, MMP-8, ICTP, and Pg in the diagnosis of periodontal disease [307]. Hu et al. (2008) [309] conducted a study to identify protein biomarkers in human saliva for diagnosing OSCC. The researchers obtained whole saliva samples from OSCC patients healthy individuals and discovered differential expression of salivary proteins between the OSCC patients and healthy individuals. The five candidate cytokine biomarkers that were successfully validated included IL-1ra, IL-6, IL-8, MIP-1α, and MIP-1β, known to be involved in inflammation, angiogenesis, chemotaxis, and cell proliferation [309].

Metabolites are another type of salivary biomarkers used for diagnosing oral diseases. Wei et al. (2011) [310] employed ultraperformance liquid chromatography coupled with quadrupole/time-of-flight mass spectrometry (UPLC-QTOFMS) to investigate the salivary metabolomics profiles OSCC, oral leukoplakia (OLK), and healthy subjects. The authors reported that valine, lactic acid, and phenylalanine effectively distinguish OSCC from the controls or OLK [310]. On the other hand, a recent study utilized GC-MS to analyze variation in salivary metabolites in OSCC and OLK patients in comparison to controls [311]. The authors reported significant differences in the levels of 15 metabolites among OSCC, OLK, and control groups (Table 2) [311]. The study findings propose the potential use of salivary metabolomics as a promising tool for identifying tumor-specific biomarkers, aiding in the early diagnosis and prediction of OSCC and oral leukoplakia [311]. Likewise, in South American patients with OSCC, GC-MS analysis revealed upregulation of malic acid, protocatechuic acid, and 2-ketoadipic acid in OSCC patients, whereas maltose, lactose, and catechol levels were reduced in these patients, thus suggesting alterations in metabolite levels reflect the distinct metabolic pathways affected in OSCC [312]. In addition to the techniques mentioned above, polymer spray ionization mass spectrometry (CPSI-MS) was used to measure the levels of various salivary metabolites associated with OSCC [313]. Differential expression of metabolites was reported in OSCC samples, while MMP-9, chemerin, glycine, proline, citrulline, and ornithine exhibited higher levels compared to the control group, choline, betaine, pipecolinic Acid, and I-Carnitine were found to be lower in OSCC compared to the control group [313]. Vis-à-vis, other specialized techniques, including reverse phase liquid chromatography with mass spectrometry (RPLC-MS), hydrophilic interaction chromatography-MS (HILIC-MS), capillary electrophoresis-time-of-flight MS (CE-TOFMS), nuclear magnetic resonance spectroscopy, direct flow injection/liquid chromatography mass spectrometry, inductively coupled plasma mass spectrometry, and high performance liquid chromatography have helped to identify and quantify distinct salivary metabolites in OSCC patients compared to healthy controls [300, 303, 314–317].

It should be noted that studies of saliva carried out by different scientific groups using different analytical methods have identified different sets of differential metabolites and different metabolic pathways affected by the disease. However, the consensus is that the most affected pathways in OSCC patients are glycolysis (Warburg effect) [269, 273, 275, 277], choline metabolism [267, 268, 272], urea cycle [267–270, 275, 277], and TCA cycle [269, 273, 275, 277]. Metabolites associated with these pathways show the largest fold change with the highest statistical significance and can be considered as potential biomarkers for early diagnosis of OSCC [268, 269, 272, 273, 277].

Salivary diagnostics for OSCC can potentially serve as a valuable tool for population screening, monitoring patients at risk of recurrent tumors, and ultimately enhancing the survival rate of individuals with this disease [318].

#### Blood: serum and plasma

Aside from saliva, blood is also frequently employed in metabolomic-based studies [298]. Both plasma and serum encompass a rich variety of metabolites, and ongoing research indicates that the metabolite content within the aqueous phase is comparable between plasma and serum [298]. Notably, alterations in the chemical and protein metabolic composition in blood samples collected from individuals with various pathologies or diseases, including cancer, can be detected [298].

NMR spectroscopy on blood samples from oral cancer patients was reported to exhibit a unique profile of disrupted energy metabolism in blood serum, characterized by changes in lipolysis (resulting in ketone bodies accumulation), perturbations in the TCA cycle, and alterations in amino acid catabolism [319]. This study also revealed the metabolite profile linked to OSCC; choline, along with betaine, dimethylglycine, carnitine, and acetyl-carnitine, aids in providing a clear distinction between early and late-stage OSCC [319]. Similarly, another study identified glutamine, propionate, acetone, and choline as potential biomarkers to distinguish OSCC from OLK [320]. Metabolomic analysis of serum from HNSCC patients using GC-MS revealed higher levels of various metabolites associated with the glycolytic pathway, including glucose, while the levels of several amino acids were lower [321]. Notably, Yang and colleagues [322] aimed to develop panels of serum metabolite markers with high sensitivity and specificity for OSCC screening and diagnosis using conductive polymer spray ionization mass spectrometry (CPSI-MS). Through cohort analysis utilizing CPSI-MS in serum, the authors identified the presence of histidine metabolism, arginine and proline metabolism, sphingolipid metabolism, and aminoacyl-tRNA biosynthesis, potential clinical markers for indicating the onset of OSCC tumorigenesis [322]. Surprisingly, the combination of CPSI-MS with an orthogonal partial least square-discriminant analysis (OPLS-DA) model effectively distinguished between (T1, T2) and (T3, T4) stages, suggesting a promising potential of CPSI-MS/ML as a practical, rapid, and cost-effective method for both screening and diagnosing OSCC [322]. Bag and colleagues [323] investigated the changes in choline metabolism in OSCC using (1)H and (13)C NMR analysis of serum samples from patients and healthy controls. While they reported loss of choline, its breakdown product, trimethylamine N-oxide, was increased in OSCC as compared to healthy controls, indicating abnormal choline metabolism [323]. In addition, they reported elevated levels of the metabolite malonate in OSCC, indicating trimethylamine N-oxide and malonate as crucial metabolic signatures for oral cancer, particularly in cases without a prominent Warburg effect [323]. Quadrupole time of flight-liquid chromatography–mass spectrometry compared the serum metabolites of patients with OSCC and OKL and reported significant increase in substantial upregulation of putrescine, 8-hydroxyadenine, and 5,6-dihydrouridine in OSCC compared to OKL, suggesting their potential utility in predicting the malignant transformation of oral leukoplakia [324].

A very recent study was conducted by Polachini and colleagues [325] using NMR and MS techniques to evaluate the plasma metabolic profile of OSCC patients and controls. The findings of the study report a unique plasma metabolic profile associated with OSCC, suggesting abnormalities in ketogenesis, lipogenesis, and energy metabolism, which were more pronounced in advanced stages of the disease, potentially contributing to inflammation, suppressing immune responses, and fostering tumor growth [325]. Plasma metabolites were analyzed in OSCC, and oral erosive lichen planus (OELP) was performed using UHPLC-Q-HRMS; a diagnostic panel comprising decanoylcarnitine, cysteine, and cholic acid was identified for OSCC diagnosis [326]. Metabolites such as uridine, taurine, glutamate, citric acid, and LysoPC(18:1) were identified as potential biomarkers indicating the malignant transformation of OELP to OSCC [326]. In summary, blood metabolomics reveals the pathways that are most affected by salivary metabolomics, i.e., energy metabolism, TCA cycle, choline metabolism, and metabolism of certain amino acids.

#### Urine

Although urine samples are used in metabolomic studies [299], akin to other biofluids, there is only one reported study specific to oral cancer using urine in OSCC metabolomic studies. Xie et al. (2012) [327] performed GC-MS in urine samples of patients with OSCC and OLK as well as healthy donors. While metabolites like valine and 6-hydroxynicotic acid discriminated OSCC from healthy donors, metabolites such as 6-hydroxynicotic acid, cysteine, and tyrosine provided a distinguishment between OSCC and OLK [327]. Additionally, a recent study identified 30 urine metabolites significantly associated with metabolomic alterations in humans who chew smokeless tobacco using targeted LC-ESI-MS/MS metabolomics methodology [328]. In laryngeal cancer, urine metabolomics using reversed-phase liquid chromatography coupled with quadrupole time-of-flight mass spectrometry (RPLC-QTOF/MS) determined six metabolites (D-pantothenic acid, palmitic acid, myristic acid, oleamide, sphinganine and phytosphingosine) associated in laryngeal cancer [329].

Nevertheless, the utilization of urine samples for OSCC metabolomics necessitates additional validation through additional independent studies.

### Tissue samples

Rather than assessing metabolites in fluids as an indirect reflection of OSCC characteristics, numerous studies have concentrated on analyzing metabolic variations within tumor tissues. Researchers have identified metabolites in tissue specimens, revealing associations between these metabolites and tumor invasion, neuropathic pain, and lymph node metastasis [330, 331].

High-resolution magic-angle spinning (HR-MAS) proton NMR spectroscopy in HNSCC and lymph-node metastatic tissues showed elevated levels of lactate, amino acids, choline-containing compounds, creatine, taurine, glutathione, and decreased levels of triglycerides associated with highly active glycolysis, increased amino acids influx, altered energy metabolism, membrane choline phospholipid metabolism, and oxidative and osmotic defense mechanisms [332]. Similarly, in OSCC, proton high-resolution magic angle spinning magnetic resonance (HR-MAS MR) spectroscopy revealed that OSCC malignant tissues had elevated levels of glutamate, choline, phosphocholine, lactate, acetate, taurine, glycine, leucine, lysine, isoleucine and alanine, and lower levels of creatine and PUFA involved in lipidogenesis, protein synthesis, and volume regulation during tumor progression [333]. This study showed that proton HR-MAS MR spectroscopy proves effective in discerning metabolic alterations between malignant and non-malignant tissues [333]. Furthermore, Young et al. (2019) [334] developed a panel of metabolites based on the GC-MS untargeted and UHPLC-MS/MS targeted metabolic and revealed four amino acids as negative margin markers and six amino acids as dysplastic margin markers in OSCC. In addition, while one study successfully differentiated between cancer and stromal regions in OSCC utilizing imaging MS [335], another study employed DESI-MS imaging to create 14 lipid ion molecular diagnostic models, aiding in the determination of safe surgical resection distances for OSCC [336]. GC-MS-based metabolite profiling, coupled with comprehensive chemometric analysis of oral cancer tissue samples, identified biomarker metabolites, including amino acids and fatty acids, that can significantly distinguish oral cancer from control groups [337]. Dickinson and colleagues used mass spectrometry-based lipidomics to analyze lipid species, classes, and glycerophospholipid fatty acid species in paired tumor and healthy tissue samples from OSCC patients and reported that OSCC tissue shows altered lipid composition and metabolism compared to normal oral tongue mucosa [69].

In the interest of applying metabolomics in clinical practice and precision medicine, several significant limitations emerge, hindering its widespread application, particularly in regard to the validation of biomarkers derived from metabolomic analysis [338, 339]. These challenges encompass both technical and logistical aspects, further impacting the reliability and reproducibility of metabolomic findings [340, 341]. One notable limitation lies in the inherent complexity and variability of metabolomic data, influenced by several factors, including diet, lifestyle, and environmental conditions [341]. This variability can complicate the establishment of reliable biomarkers and hampers the reproducibility of results across different studies and populations [338, 340]. Additionally, standardization issues in sample collection, processing, and analysis further contribute to the lack of reliability and comparability [338, 340]. Overcoming these challenges necessitates the development and implementation of standardized protocols and methodologies across the metabolomics group [340, 341]. Furthermore, technological limitations, including sensitivity and specificity constraints in current analytical techniques, can hinder accurate detection and quantification of metabolites, plausibly leading to the exclusion of clinically relevant markers [338, 339]. To address these limitations, ongoing advancements in analytical technologies are vital in addition to improving sensitivity, specificity, and throughput [338, 339]. Moreover, the vast amount of data generated by metabolomic analyses demands sophisticated bioinformatics tools and expertise for consequential interpretation, thus complicating the differentiation between causative and correlative metabolic changes and poses as a barrier to extract biologically and clinically relevant information [340, 341]. To mitigate this limitation, there is a need for continual investment in bioinformatics research and the development of user-friendly analytical platforms to allow researchers to easily access these platforms across disciplines [340, 341]. Another vital aspect is the intricate and lengthy metabolic biomarker validation pipeline, which demands extensive clinical validation and regulatory approval [338, 340]. To simplify this challenge, collaborative efforts amongst researchers, clinicians, and industry stakeholders are necessary to establish robust validation frameworks and enhance metabolic discoveries from the translation level into clinical practice [338, 340]. Finally, interdisciplinary collaboration is critical for directing the multifaceted challenges of metabolomics in precision medicine, demanding ongoing engagement amongst biologists, chemists, bioinformaticians, and clinicians [338, 340]. Thus, by fostering a concerted ecosystem and implementing standardized methodologies, leveraging technological advancements and robust data analytical tools, the limitations of metabolomics can be subdued, thus unlocking its full potential to revolutionize precision medicine and improve clinical outcomes.

## Therapeutic agents targeting metabolism in OSCC

Early intervention in metabolism has the potential to impede or decelerate the emergence of drug resistance. Additionally, the distinct metabolic response of normal cells to drugs remains unclear. By contrast with normal cells, the unique metabolites, or metabolic pathways of tumor cells in response to various drugs, can serve as targets for metabolic interventions in cancer therapy. Metabolomics profiling proves valuable in delineating the metabolic dynamics of tumor cells exposed to different therapeutic agents.

Metformin, commonly prescribed for type II diabetes, has been acknowledged for its antitumor activity, exerted through various mechanisms [342]. By inhibiting the mTOR pathway, metformin not only restricts protein synthesis and gluconeogenesis but also enhances fatty acid oxidation and induces cell cycle arrest and apoptosis, thus contributing to its antitumor effects [343, 344]. While STF-31 is a characterized inhibitor of GLUT1 [345], Glutor selectively inhibits glycolytic flux by targeting GLUT1, GLUT2, and GLUT3 [346]. Benitrobenrazide, a HK2 selective inhibitor, demonstrated efficiency in immunocompromised mice [347]. Telaglenastat stands as a pioneering oral glutaminase inhibitor, disrupting glutamine consumption by tumors [348]. By impeding this process, telaglenastat elevates glutamine levels in the tumor microenvironment, leading to heightened immune cell activity [348]. Similarly, numidargistat, a small-molecule arginase inhibitor, inhibits arginase, to restore the cellular proliferation and cytotoxic activity of activated T cells, promoting a more effective immune response against cancer [349]. On the other hand, the bioengineered human PEGylated arginase 1 enzyme, pegzilarginase is designed to degrade arginine into ornithine and urea, effectively reducing blood arginine levels, thus potentially influencing the growth and survival of cancer cells [350]. Likewise, several molecules, such as cifrodenant, imaradenant, taminadenant, and etrumadenant inhibit adenosine signaling [351–354]. Therapeutic interventions targeting metabolic pathways in OSCC are tabulated in Table 4.

**Table 4:** Experimental therapeutic drugs targeting potential metabolic pathways involved in OSCC

Glucose Metabolism			
Drug	Target Gene/Pathway	Mechanism Involved	Reference
Metformin	mTOR pathway	• Inhibits protein synthesis and gluconeogenesis. • Promotes fatty acid oxidation, cell cycle arrest, and apoptosis.	[343, 344]
Polydatin	G6PD	• Accumulation of ROS. • Induces apoptosis.	[366]
Glutor	GLUT1, GLUT2, and GLUT3	• Inhibits glycolytic flux.	[346]
Silybin	GLUT, EGFR	• Promotes G1 cell cycle arrest. • Inhibits glycolysis and angiogenesis.	[367]
Lonidamine	Hexokinase	• Inhibits glycolysis.	[368]
Melatonin	MTHFD1L, CREB1, formate	• Inhibits glycolysis and cell proliferation. • Induces OXPHOS, ROS production, apoptosis, and mitotic phagocytosis.	[369, 370]
**Lipid Metabolism**			
Terbinafine and butenafine	Squalene epoxidase	• Promotes sensitivity of cancer cells to drugs.	[371, 372]
Anti-CD36 neutralizing antibodies (FA6.152 and JC63.1)	CD36	• FA6.152 inhibits CD36 interactions with thrombospondin, collagens, and fatty acids. • JC63.1 blocks fatty acid and oxidized low-density lipoprotein uptake. • Inhibits metastasis.	[373]
Artesunate	Nrf2–ARE pathway	• Induces ferroptosis.	[94]
**Amino-Acid Metabolism**			
Telaglenastat	GLS	• Increases Gln levels. • Promotes immune cell activity.	[348]
IPN60090	GLS	• Inhibits cell proliferation.	[374]
Combination of CPI-613 and CB-839 (GLS1 inhibitor)	GLS1, PDH and α-KGDH	• Inhibits cancer cell multiplication. • Inhibits the metabolic dependence of HNSCC cells on Gln • Increases sensitivity of cancer cells.	[375]

Chen et al. (2022) [355] recently performed comparative metabolomics to analyze the divergent metabolic reactions of oral cancer cells and normal oral epithelial cells following exposure to cisplatin to pinpoint the metabolites serving as markers for early responses in oral cancer cells. The study identified glyoxylate and dicarboxylate metabolism, along with fructose, malate, serine, alanine, sorbose, and glutamate, as specific enriched metabolic pathways and biomarkers indicative of oral cancer cells response to cisplatin [355]. However, further investigation is warranted to elucidate the mechanism and significance of early serine elevation induced by cisplatin in oral cancer cells [355].

Targeting epigenetic alterations (DNA methylation, histone modifications, chromatin remodeling, and non-coding RNAs) using inhibiting enzymes such as DNMTs and HDACs have demonstrated a promising approach for therapy. CpG methylation alterations have the potential to be targeted for epigenetic therapies in OSCC. Zebularine, a DNMT inhibitor, reduced the growth of OSCC cells [356]; however, in combination with cisplatin, it significantly induced apoptosis, further inhibiting tumor growth [357]. Other epigenetic inhibitors such as 5-azacytidine (5-aza-CR) and 5-aza-2′-deoxycytidine (5-aza-CdR or decitabine) can inhibit DNMTs; in OSCC cells, 5-aza-CdR was found to reverse methylation and restore the expression of p16INK4a [358]. As mentioned in Table 2, methylation of the MGMT gene is associated with OSCC; O6-benzylguanine (O6-BG), a potent inhibitor of MGMT, was found to enhance the anti-tumor efficacy of 5-FU in OSCC cells [359]. Histone deacetylase (HDAC) inhibitors prevent histone deacetylation, thus promoting the regulation of genes associated with cell survival, proliferation, differentiation, and apoptosis [360]. A few of the HDAC inhibitors used as therapeutics against OSCC are mentioned in the table below (Table 5).

**Table 5:** HDAC inhibitors used as experimental therapeutic interventions in OSCC

HDAC Inhibitor	Target Gene	Mechanism of Action	Reference
Trichostatin A	p21^WAF1^, CREB-binding protein, CyclinE, Cyclin A, Bak and Bax, E2F-1, E2F-4, HDAC1, p53, Rb	• Inhibits cell growth. • Promotes cell cycle arrest and apoptosis.	[376–378]
Phenylbutyrate	TNF-α	• Enhances DNA repair and cell survival. • Reduces oxidative stress.	[379]
Sodium Butyrate	CDK6, p21^WAF1^, p27, CDK2, Rb, Cyclin D1, Cyclin B1 and Cyclin E	• Inhibits cell proliferation. • Promotes G1 and G2/M cell cycle arrest.	[380, 381]
Romidepsin	Mapsin and hTERT	• Promotes G1 and G2/M cell cycle arrest. • Inhibits cell invasion and angiogenesis.	[382, 383]
Entinostat	p21, acetylation histones H3 and H4	• Inhibits tumor growth and proliferation. • Promotes G0/G1 cell cycle arrest. • Induces oxidative stress and apoptosis	[384, 385]
Apicidin	HDAC8	• Inhibits tumor growth and proliferation. • Induces autophagy and apoptosis	[386]

On the contrary, studies have also reported inhibition of glycolysis, lipid metabolism as well as epigenetic modifications by natural compounds or synthetic vitamin derivatives may serve as sensitizers for apoptosis in cancer cells, acting in conjunction with adjuvant therapies in OSCC [361–365].

## Conclusion

In conclusion, recent advancements encompassing metabolomics to single-cell methodologies have significantly broadened the research landscape, establishing cancer metabolism as a vibrant and prolific domain within cancer biology. Although metabolomics is less utilized than other omics approaches, it holds substantial potential to impact key aspects of oncology, including screening, diagnosis, and therapy. Current knowledge about which metabolites can reflect OSCC status can allow us to improve the metabolic alterations in OSCC by adopting a healthier lifestyle, such as losing weight, quitting smoking, and reducing alcohol consumption. Further, targeted therapies for addressing epigenetic alterations and genetic mutations in OSCC offer the potential to modulate metabolic pathways and networks within cancer cells. The correction of these metabolic alterations in OSCC opens the possibility of developing innovative treatment options for a disease that is typically associated with a poor prognosis. Unlike other omics technologies, metabolomics cannot be completely comprehensive with a single approach. To maximize its utility, combining metabolomics with other omics approaches and hypothesis-driven investigations is currently recommended to unveil functionally and diagnostically relevant alterations in cancer cells, including OSCC. With ongoing advancements in standardized protocols and user-friendly analysis platforms, metabolomics is poised to play an increasingly significant role in cancer research.

## Data Availability

Not applicable.
